# Improving the care for people with acute low-back pain by allied health professionals (the ALIGN trial): A cluster randomised trial protocol

**DOI:** 10.1186/1748-5908-5-86

**Published:** 2010-11-10

**Authors:** Joanne E McKenzie, Denise A O'Connor, Matthew J Page, Duncan S Mortimer, Simon D French, Bruce F Walker, Jennifer L Keating, Jeremy M Grimshaw, Susan Michie, Jill J Francis, Sally E Green

**Affiliations:** 1 School of Public Health and Preventive Medicine, Monash University, Melbourne, Australia; 2 Centre for Health Economics, Faculty of Business and Economics, Monash University, Melbourne, Australia; 3 Primary Care Research Unit, The University of Melbourne, Melbourne, Australia; 4 School of Chiropractic and Sports Science, Faculty of Health Sciences, Murdoch University, Western Australia; 5 Department of Physiotherapy, Monash University, Melbourne, Australia; 6 Clinical Epidemiology Program, Ottawa Health Research Institute, Ottawa, Canada; 7 Department of Medicine, University of Ottawa, Ottawa, Canada; 8 Department of Psychology, University College London, UK; 9 Health Services Research Unit, University of Aberdeen, Scotland, UK

## Abstract

**Background:**

Variability between clinical practice guideline recommendations and actual clinical practice exists in many areas of health care. A 2004 systematic review examining the effectiveness of guideline implementation interventions concluded there was a lack of evidence to support decisions about effective interventions to promote the uptake of guidelines. Further, the review recommended the use of theory in the development of implementation interventions. A clinical practice guideline for the management of acute low-back pain has been developed in Australia (2003). Acute low-back pain is a common condition, has a high burden, and there is some indication of an evidence-practice gap in the allied health setting. This provides an opportunity to develop and test a theory-based implementation intervention which, if effective, may provide benefits for patients with this condition.

**Aims:**

This study aims to estimate the effectiveness of a theory-based intervention to increase allied health practitioners' (physiotherapists and chiropractors in Victoria, Australia) compliance with a clinical practice guideline for acute non-specific low back pain (LBP), compared with providing practitioners with a printed copy of the guideline. Specifically, our primary objectives are to establish if the intervention is effective in reducing the percentage of acute non-specific LBP patients who are either referred for or receive an x-ray, and improving mean level of disability for patients three months post-onset of acute LBP.

**Methods:**

The design of the study is a cluster randomised trial. Restricted randomisation was used to randomise 210 practices (clusters) to an intervention or control group. Practitioners in the control group received a printed copy of the guideline. Practitioners in the intervention group received a theory-based intervention developed to address prospectively identified barriers to practitioner compliance with the guideline. The intervention primarily consisted of an educational symposium. Patients aged 18 years or older who visit a participating practitioner for acute non-specific LBP of less than three months duration over a two-week data collection period, three months post the intervention symposia, are eligible for inclusion. Sample size calculations are based on recruiting between 15 to 40 patients per practice. Outcome assessors will be blinded to group allocation.

**Trial registration:**

Australian New Zealand Clinical Trials Registry ACTRN12609001022257 (date registered 25^th ^November 2009)

## Background

Dozens of evidence-based clinical practice guidelines (CPGs) are published annually with the potential to improve the quality and safety of health care. However, failure to implement CPG recommendations has resulted in patients receiving care that is inappropriate, unnecessary, or even harmful [1-3]. Effective interventions are needed to address the barriers to change required for successful implementation of CPGs.

The most comprehensive systematic review to date identified small to moderate effects of most interventions to implement CPGs [4]. Despite some promising interventions, existing studies provide limited guidance on the factors that moderate the effects of these interventions in different settings, professional groups, and for different targeted behaviours. Consequently, we know little about how to formulate effective strategies for implementing CPGs. The use of theory has been advocated to inform intervention development with the potential to improve our understanding of why a particular intervention may be effective [5,6]. Theory can provide an explicit basis for identifying determinants of change and intervention techniques to modify these determinants. This approach is supported by the United Kingdom (UK) Medical Research Council (MRC) framework for developing and evaluating complex interventions [7]. Our protocol focuses on the last phase of this framework: assessing effectiveness, cost-effectiveness, and understanding change processes [8].

Low back pain (LBP) is a common and costly problem. At any one time, 26 in every 100 Australians have LBP, and 79% of Australians will experience it at some time in their lives [9]. The direct and indirect cost of LBP in 2001 was estimated to total AUD 9,175 million [10]. In 2003, the Australian National Health and Medical Research Council (NHMRC) endorsed a CPG for acute LBP [11] that provided evidence for the diagnosis, prognosis and treatment of acute non-specific LBP. The relevant key messages are: that plain x-rays of the lumbar spine are not routinely recommended for people with acute non-specific LBP because they are of limited diagnostic value and provide no benefits in pain, function, or quality of life [11]; and advising these patients to stay active produces a beneficial effect on pain, rate of recovery, and function when compared to bed rest and a specific exercise regimen (based on level I evidence) [11]. While the NHMRC CPG was published in 2003, recent systematic reviews of randomised trials still support these two recommendations [12-14].

The CPG was disseminated in 2004 via: distribution of an evidence summary booklet to 40,000 practitioners in Australia, including allied health practitioners; distribution of consumer information sheets; and publicising the availability of materials on the NHMRC website. It is likely, therefore, that most practitioners who manage people with LBP will be aware of and have access to the CPG. However, recent research in Australian general practice indicates that patterns of LBP management (particularly in relation to the x-ray and advice to stay active behaviours) by general practitioners (GPs) have remained unchanged [15]. To our knowledge, the effect that dissemination of the CPG has had on Australian allied health practitioners' behaviour has not been studied.

There is a need to establish effective methods for facilitating the uptake of CPGs in allied health. About one-third of the care for LBP in Australia is provided by physiotherapists and chiropractors [16], however research suggests that care is not always consistent with the CPG recommendations. International studies demonstrate that chiropractors often investigate people with acute non-specific LBP using plain radiography [17-23]. Physiotherapy management is less likely to include referral for plain x-ray, however Australian physiotherapists' self-reported use of x-rays for acute non-specific LBP is still too frequent [22], and physiotherapists feel uncertain regarding the value of CPGs [24,25].

A number of randomised trials have investigated the effectiveness of interventions to increase compliance with CPG recommendations for the management of LBP by GPs [26-38]. However, there has been little equivalent research in allied health settings [39], with only three cluster randomised trials (CRTs) reported -- one in the Netherlands [40,41], and two in the UK [42,43]. The Netherlands CRT evaluated the effectiveness of an intervention consisting of two interactive workshops designed to address barriers to implementation [40,41]. Practitioners in the intervention group followed the CPG more frequently (odds ratio (OR) 2.05; 95% confidence interval (CI) 1.15 to 3.65); however, this did not result in better patient outcomes. A UK CRT assessed the effectiveness of an evidence-based, local opinion leader-led LBP educational program compared to receiving in-service training on knee pathology management and found no evidence of a difference in the odds of practitioners rating 'providing advice to return to normal activities' in the top five most important treatments delivered to acute LBP patients [42]. However, the confidence interval was wide and did not exclude potentially important effects. Another UK CRT compared practitioners receiving a printed information package providing evidence-based recommendations to no intervention [43]. Practitioners in the intervention group were more likely to record providing advice to stay active in response to a single clinical vignette (OR 1.29; 95% CI 1.03 to 1.61). However, these studies did not investigate mechanisms of action of the interventions, limiting the extent to which it is possible to explain their findings using theories of behaviour change.

We have recently completed a CRT investigating the effectiveness of a theory-based intervention for implementing the NHMRC CPG in general practice (the IMPLEMENT trial) [33]. The current trial (the ALIGN trial) extends this work to allied health. At the writing of this protocol (March to May 2010), baseline data collection, and the physiotherapy and chiropractic interventions have taken place. Data collection of the patient participants has just begun.

### Trial objectives

The primary objective of the ALIGN trial is to estimate the effectiveness of a theory-based intervention that aims to increase physiotherapists' and chiropractors' (in Victoria, Australia) compliance with a CPG for acute LBP, compared with providing practitioners with a printed copy of the guideline. Specifically, our primary objectives are to establish if the intervention is effective in:

(i) Reducing the percentage of acute non-specific LBP patients who are either referred for an x-ray, or receive an x-ray (practitioner behaviour);

(ii) Improving the mean level of disability for patients three months post-onset of an episode of acute non-specific LBP (patient level health outcome).

Secondary objectives include estimating the effects of the intervention for secondary outcomes in the following categories: (i) practitioner behaviour (provided advice to stay active, advised bed rest, referred for imaging excluding x-ray), (ii) predictors of practitioner behaviour (practitioner intention to behave in a manner consistent with the CPG's recommendations, behavioural constructs (*e.g.*, knowledge, beliefs about capabilities)), (iii) patient health outcomes (pain severity, health-related quality of life (HRQoL)), (iv) patient health behaviour (x-ray occurred), and a (v) predictor of patient health behaviour (patients' fear-avoidance beliefs (FAB)). In addition, we will assess cost-effectiveness based on the practitioner and patient-level outcomes described above, as well as assessment of HRQoL, health service utilisation, and productivity gains/losses.

## Methods

### Trial design

The design of the trial will be a cluster randomised trial, with the clusters being physiotherapy or chiropractic practices including one or more practitioners. There are challenges to employing a cluster randomised design compared to patient randomised trials. These include increased sample sizes, with associated additional costs and complexity, and increased risk of selection bias at the patient level [44,45]. Selection bias has been shown to occur in cluster trials from selective recruitment of participants by individuals who have knowledge of the allocation status [46]. Despite these challenges, randomisation at the practice level has the benefit of reducing potential contamination occurring from practitioners concurrently managing intervention and control patients, as would occur in a patient randomised trial [47-49], and practitioners within the same practice receiving different interventions, as would occur if practitioners were randomised. The latter 'contamination' may be seen as beneficial when randomisation occurs at the practice level, because this may result in greater diffusion of the intervention from interactions between practitioners [50]. Therefore in the ALIGN trial, randomisation at the practice level minimises the potential for contamination between intervention and control groups, while increasing the potential for diffusion of the intervention within intervention practices.

### Eligibility and recruitment

#### Recruitment of physiotherapy and chiropractic practices

Sampling frames of physiotherapy and chiropractic practitioners were created from lists provided by their respective Victorian Registration Boards. Updated contact details and information on their practices were obtained from the national telephone directory, an internet version of the Yellow Pages^®^ 2009. Practitioners who were unlikely to be eligible (*e.g*., those practising in tertiary hospitals or practices obviously not providing care for acute LBP patients) were removed from the sample frame. All remaining practitioners were approached and invited to participate (2,463 physiotherapists and 1,196 chiropractors). Practitioners were sent an invitation letter, explanatory statement, and consent form. Those who did not respond were sent a maximum of four reminder letters, each three weeks apart. When one practitioner in a practice agreed to participate, a list of all practitioners employed at that practice was created, and invitation letters were sent to the other practitioners informing them that the practice was included, encouraging them to participate, and allowing them to object to the practice participating if they wished. If the latter occurred, we planned to contact the practice manager for direction on whether the consenting practitioners within the practice could participate.

To increase the awareness of the trial, notices were placed in professional newsletters and the trial was promoted at conferences. Strategies to promote participation in the trial included offering professional development points (for chiropractors in the intervention group only), payment for assistance in accessing clinical files of patients with acute LBP (AUD 5 per patient), and entry into a draw to win a prize to attend a professionally relevant conference in Australia (up to a maximum of AUD 800). In addition, practitioners were advised that some randomly selected participants would have the opportunity to discuss LBP management with colleagues and experts in the field.

We initially intended to recruit 136 practices (68 physiotherapy and 68 chiropractic practices), but received a better than expected response with 210 practices wishing to participate. This included 133 physiotherapy practices with 180 physiotherapists, and 77 chiropractic practices with 88 chiropractors. Given the uncertainty in the parameters informing our sample size calculation (*e.g*., intra-cluster correlation coefficient (ICC), see 'Sample size' section), we made a decision to include all practices.

#### Recruitment of patient participants

Practitioners will recruit patient participants over a two-week period at least three months post the intervention symposia. Practices will be provided with study explanatory statements to distribute to all patients over this period. At each consultation over this period, practitioners will complete a de-identified patient encounter form recording, at a minimum, the date and start time of the encounter. The practitioner will provide a brief explanation about the study and will ask each patient for verbal consent to complete a checklist about their visit. If the patient consents, the practitioner will record basic demographic information (date of birth, sex, post code) and will determine if the patient meets the trial's inclusion criteria. Consenting patients with acute non-specific LBP who also meet other eligibility criteria will be considered patient participants. For these patients, the practitioner will complete a checklist of diagnostic procedures and interventions they have ordered, undertaken, or recommended. At the conclusion of the consultation, the practitioner will provide patient participants with additional documentation regarding further participation in the trial if they wish.

Patient participants will be provided with a brief checklist to complete and a blank envelope. The checklist asks what diagnostic procedures and interventions they have received, and also invites them to participate further in the trial by consenting to additional data collection, either through completion of a survey at three months post-onset of acute LBP, or allowing their clinical file to be audited by the research team, or both. Patient participants will be asked by the practitioner to complete the checklist before leaving the practice, place it in the provided envelope, seal the envelope, and put it in the box at reception. Those who are unable to complete the checklist at the time of their consultation will be given the option of posting the checklist directly to the research team. Patient participants will be informed that their care will not be affected by their participation in the trial, and that the information they provide will not be available to practitioners at the practice. Patient participants will remain de-identified, unless they consent to additional data collection (completion of survey or file audit, or both). To promote participation in additional data collection, patients will be offered the incentive of being entered into a draw to win a mobile phone.

The patient recruitment process has the potential to introduce selection bias because patients are recruited post-randomisation, and this is undertaken by practitioners who are aware of their own allocation status [51]. In cluster trials such as ALIGN, which include patients with an acute condition, it is generally not possible to recruit patient participants prior to randomisation of practices [45]. Potential strategies for minimising selection bias occurring through patient recruitment in ALIGN included (i) researchers blinded to group allocation recruiting in practices, (ii) reception staff recruiting patients, (iii) researchers blinded to group allocation searching clinical files without patient consent, (iv) using de-identified data produced by software for trawling electronic clinical files, and (v) using administrative data. It was not possible for us to implement any of these options. We did not have the resources available to place recruiters at each of the practices (option (i)). Some practices did not have reception staff, precluding the use of option (ii). Australia's state and commonwealth privacy legislation does not allow researchers to access clinical files without patient consent (option (iii)) [52]. Most physiotherapy and chiropractic practices do not use electronic clinical files, precluding the use of option (iv). Finally, unlike in the general practice setting where administrative data on x-ray referral is available (MediCare Australia), this data is not available for physiotherapists and chiropractors. Given these limitations, we plan to report data to assess potential selection bias. Details are available in sections 'Analysis subsets' and 'Descriptive analyses at baseline.'

#### Inclusion criteria

Physiotherapy and chiropractic practices were included if the following criteria were met:

1. At least one practitioner within the practice provided written informed consent.

2. The practice was located in the state of Victoria, Australia.

Physiotherapists and chiropractors were included if they provided written informed consent and practised within one of the participating practices.

Patient participants will be included if the following criteria, determined by the practitioner, are met:

1. They attend a consenting practitioner for acute non-specific LBP (of duration less than three months).

2. Provide consent.

3. Are 18 years of age or older.

4. Are able to understand and read English.

#### Exclusion criteria

Physiotherapy and chiropractic practices were excluded if the practice manager objected to any practitioners within the practice participating in the trial.

Physiotherapists and chiropractors were excluded if any of the following criteria were met:

1. They were investigators of the trial.

2. They were members of the ALIGN advisory committee.

3. They were involved in interviews that informed the development of the intervention.

4. They worked at more than one of the included practices in the trial.

Patients attending the enrolled practices will not be eligible for inclusion if any of the following criteria are met. These criteria reflect the clinical scope of the acute LBP NHMRC CPG [11].

1. Radicular leg pain is present; this is defined as leg pain described as shooting, lancinating, or electric in quality, extends below the knee, has a dermatomal distribution with or without paraesthesia.

2. They have had previous spinal surgery.

3. 'Red flags' are present alerting the possibility of serious conditions such as malignancy, infection, or fracture.

4. They are pregnant.

### Randomisation and allocation concealment

Practices meeting the inclusion criteria were randomly allocated at the same time to receive either the intervention or control. Restricted randomisation was used to reduce the probability of baseline imbalance in potential confounding variables, and to provide greater statistical power [50]. Four strata were defined by professional group (physiotherapists and chiropractors) and the location of the practice (rural or metropolitan; defined from the Rural, Remote, and Metropolitan Areas classification system [53]). Within stratum, practices were allocated to the intervention and control groups with equal probability (1:1 randomisation ratio). This was achieved by generating a computer random number (in the statistical software package Stata [54]) for each practice, sorting on the random number within each stratum, and allocating every alternate practice to the intervention group.

Professional group was considered an important stratification variable, because management of patients with acute non-specific LBP may vary because of professional training and philosophy. A survey of Australian primary care physicians, including physiotherapists and chiropractors, suggested a difference in self-reported x-ray use for acute non-specific LBP between the professional groups [22]. In addition, in 2009 we undertook a survey investigating the attitudes, beliefs, and intentions of physiotherapists and chiropractors in Australia, regarding their management of patients with acute LBP (response rate = 33%, sample size = 469). Differences in intention to refer patients for x-ray and provide advice to stay active were found between the professional groups. Specifically, of the next 10 patients presenting with acute LBP, chiropractors intended to refer 3.7 (95% CI 3.2 to 4.3) more patients for an x-ray compared to physiotherapists, and provide advice to stay active to 0.6 (95% CI 0.4 to 0.9) fewer patients (Unpublished data: O'Connor D, Monash University, Australia).

We made a decision to include location of the practice as a stratification variable because the rates of x-ray referral may vary with geographical proximity to imaging centres, and because of geographic variation in socioeconomic barriers to health service utilisation, including diagnostic imaging [55].

Finally, consideration was given to stratifying by cluster size. Cluster size is quite commonly used as a stratification variable in community intervention trials [50] because the size of a cluster is often a proxy for other variables, which may be predictive of outcome, but are more difficult to measure (*e.g*., educational environment within a practice). In the ALIGN trial, we did not stratify by size of the practice because we were not able to obtain accurate information on this variable.

A statistician who was independent of the trial team undertook the randomisation. He was provided with a file containing practice codes and stratification variables. The file contained no identifying information about the practices.

### Blinding

It was not possible to blind investigators involved in the delivery of the intervention to group allocation. In addition, due to the nature of the intervention, it was not possible to blind the practitioners to group allocation. However, practitioners only received minimal information about the intervention content in the recruitment material. They were informed that they may have to access materials about management of LBP either through the Internet, or they may be invited to attend an interactive workshop. Patient participants will be informed through recruitment materials that their practitioner is participating in a study assessing practitioners' management of patients presenting with acute LBP; they will not be informed of the study design, and therefore of their practitioners' group allocation. However, because the practitioner is not blinded, it is possible that they might reveal their allocation to participating patients. Outcome assessors extracting data from clinical files of patients, and research assistants entering practitioner and patient completed checklists and questionnaires, will be blinded to group allocation. The trial statistician will not be blinded to group allocation.

### Interventions

#### Control group

Participants in control group practices received a printed copy of the summary version of the guideline and a written reminder of how to access it [11]. For some practitioners, this provides an additional exposure to the CPG that was originally disseminated by the NHMRC in 2004.

#### Intervention group

Intervention group practitioners received a theory-based implementation intervention developed to address the hypothesised determinants of CPG implementation identified in phase one of this project. In phase one, semi-structured interviews, underpinned by a theoretical domains framework [56], were conducted with physiotherapists and chiropractors in Victoria, Australia. Thematic analysis was used to map the identified barriers and facilitators of the target behaviours to the theoretical domains framework. The most salient domains identified formed the basis of a survey, which was distributed to a random sample of practising physiotherapists and chiropractors in Victoria, South Australia, and Western Australia. The survey quantified the association between constructs from these domains and intention to practice in a manner consistent with the guideline recommendations. Both the findings of interviews and survey informed the design of the intervention, and will be published elsewhere.

The intervention consisted of: a full-day symposium-style event involving a combination of didactic lectures delivered by peer opinion leaders (identified in consultation with representatives from the physiotherapy and chiropractic associations), small group discussion led by trained clinical facilitators, and practical sessions; supporting written material; and a follow-up phone call. Separate symposia were held for physiotherapists and chiropractors. All practitioners in the intervention group, including those who were not able to attend the symposium, received a DVD including videos of the didactic sessions and printed resources about LBP management. A clinical member of the project team attempted to follow-up all practitioners with a telephone call to discuss difficulties encountered in implementing behaviours and strategies to overcome these. More detail on the intervention, including the development process, will be reported in a separate publication. Symposia details are available in Additional File 1 - 'ALIGN intervention content.'

Finally, while not formally a component of the intervention or control group, the practitioner data collection procedure involves completion of patient checklists about LBP management and may act as a prompt to change practitioner behaviour. The checklist includes a broad range of diagnostic procedures and interventions potentially used for patients with acute non-specific LBP, irrespective of supporting evidence.

#### Timing of recruitment, intervention delivery, and follow-up

The physiotherapist and chiropractic symposia took place on 20 and 27 February 2010, respectively. Practitioners in the intervention group were mailed a DVD of material from the symposium for their professional group on 29 March 2010. Practitioners in the intervention group received a follow-up phone call two to four weeks after either attending the symposium or being sent the DVD. Materials were sent to the control group on 10 March 2010.

Patient participant recruitment will take place over a five-week period, beginning at least three months post-symposium delivery (31 May 2010). Each practice will recruit patients for a period of two weeks (a longer period was judged to place too great a burden on practitioners). Practices will be randomly allocated to recruit patients in either the first (31 May to 11 June 2010) or second (21 June to 2 July 2010) data collection period. Practitioners who are not able to collect data in either of these periods (*e.g*., on holiday), will be invited to select an alternative fortnight of data collection between July and September 2010.

**Figure 1:**
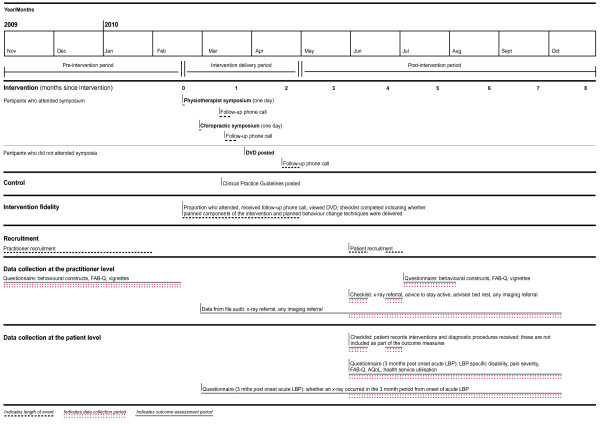
**Timing of recruitment, intervention delivery, follow-up of practitioner, and patient participants**.

Figure 1 details the timing of recruitment, intervention delivery, and data collection periods for practitioner and patient participants.

#### Intervention fidelity

We will evaluate intervention fidelity, which is the extent to which the intervention, as delivered, was adhered to as planned [57]. The proportion of practitioners in the intervention group who attended the symposium, received the follow-up call, and viewed the DVD has been documented. The symposia were audio- and video-recorded and will be transcribed verbatim. Observed adherence will be assessed by content analysis of the symposium transcripts to evaluate which intervention sections were covered, and which behaviour change techniques were delivered. An independent assessor attended each symposium and completed a checklist to indicate whether or not planned intervention components and behaviour change techniques were used. We will report the proportion of practitioners who received each different component of the intervention and the data from the checklist completed by the independent assessor in the main trial publication; the remaining components of this evaluation will be reported in a separate publication.

### Study outcomes

#### Primary outcomes

The primary outcome at the practitioner level is whether the practitioner orders, undertakes, or recommends a lumbar x-ray for the acute non-specific LBP patient over the two-week data collection period (occurring three months post-symposium). The primary outcome at the patient level is LBP specific disability three months post-onset of their acute LBP episode (Table 1).

**Table 1:** Outcome measures

Outcome *(outcome category)*	Data collection method	Outcome assessment period	Source	Level data collected at
**Inference intended at the practitioner level**				
X-ray referral *(practitioner behaviour)*^1^	Checklist completed by practitioner	3 to 4 months post-symposium	Practitioner	Patient
Advice to stay active *(practitioner behaviour)*	Checklist completed by practitioner	3 to 4 months post-symposium	Practitioner	Patient
Imaging referral excluding x-ray *(practitioner behaviour)*	Checklist completed by practitioner	3 to 4 months post-symposium	Practitioner	Patient
Advised bed rest *(practitioner behaviour)*	Checklist completed by practitioner	3 to 4 months post-symposium	Practitioner	Patient
X-ray referral (file audit) *(practitioner behaviour)*	Clinical file audit	0 to 7 months post-symposium	Practitioner case notes	Patient
Imaging referral excluding x-ray (file audit) *(practitioner behaviour)*	Clinical file audit	0 to 7 months post-symposium	Practitioner case notes	Patient
Intention to adhere to CPG recommendations:	Questionnaire	Baseline, 4 months post-symposium	Practitioner	Practitioner
X-ray referral				
Imaging referral excluding x-ray				
Advice to stay active				
Bed rest advice				
*(predictor practitioner behaviour)*				
Behavioural constructs^2 ^(e.g., knowledge, beliefs about capabilities) *(predictor practitioner behaviour)*	Questionnaire	Baseline, 4 months post-symposium	Practitioner	Practitioner

**Inference intended at the patient level**				
LBP specific disability^1^ *(health outcome)*	Questionnaire	3 months post-onset acute LBP episode	Patient	Patient
Pain severity *(health outcome)*	Questionnaire	3 months post-onset acute LBP episode	Patient	Patient
X-ray occurred *(health behaviour)*	Questionnaire	3 months post-onset acute LBP episode	Patient	Patient
Fear-avoidance beliefs *(predictor health behaviour)*	Questionnaire	3 months post-onset acute LBP episode	Patient	Patient
Health-related Quality of Life *(health outcome)*	Questionnaire	3 months post-onset acute LBP episode	Patient	Patient
Health Service Utilisation and Productivity Gains/Losses	Questionnaire	3 months post-onset acute LBP episode	Patient	Patient

Table adapted from McKenzie *et al*. [33]. See Additional File 2 - 'ALIGN outcome definitions' for details of outcome definitions.

^1^Primary outcome. ^2^Table 2 provides details of the behavioural construct domains.

The rationale for selecting these outcomes as primary outcomes is fully described in McKenzie *et al*. [33]. In brief, the intervention focused on two key recommendations from the CPG: diagnostic plain x-rays of the lumbar spine are rarely necessary in the management of acute LBP, and patients should be advised to remain active. The primary outcome at the practitioner level measures the effectiveness of the intervention in changing the practitioners' behaviour for the first recommendation, x-ray referral. The primary outcome at the patient level measures the effectiveness of the intervention in changing the practitioners' behaviour for the second recommendation, providing advice to stay active, and assessing whether this change results in improvements for the patients in terms of short-term disability.

#### Secondary outcomes

Secondary outcomes include measures of practitioner behaviour (provided advice to stay active, advised bed rest, referred for imaging excluding x-ray), predictors of practitioner behaviour (practitioner intention to behave in a manner consistent with the CPG's recommendations, behavioural constructs), patient health outcomes (pain severity, HRQoL), a predictor of patient health behaviour (FAB), and a patient health behaviour (x-ray-occurred). The outcomes, data collection methods, and assessment periods are included in Tables 1 and 2. Justification for the inclusion of these outcomes follows.

**Table 2:** Behavioural construct domains

		Domain measured for behaviour	
		
Domains	Explanation	**X-ray referral**^**1**^	Advice to stay **active**^**2**^
**Behavioural intention**			
Intention	The extent to which the practitioner intends to perform the behaviour.	✓	✓
**Other behavioural domains**			
Beliefs about capabilities	The extent to which the practitioner feels confident in/control over performing the behaviour.	✓	✓
Beliefs about consequences	The extent to which the practitioner is in favour of performing the behaviour and has positive behavioural beliefs.	✓	✓^3^
Knowledge	Whether the practitioner has knowledge of the behaviour.	✓	✓
Professional role and identity	The extent to which the practitioner feels it is their professional responsibility to perform the behaviour.	✓	✓
Social influences	The extent to which the practitioner feels social pressure to engage in the behaviour.	✓	✓
Environmental context and resources	The extent to which the practitioner feels the environmental context supports performance of the behaviour.	✓	✓
Memory	The extent to which the practitioner remembers to perform the behaviour.	✗	✓

^1^Managing patients without referral for plain x-ray.

^2^Advising patients to stay active.

^3^Includes measurement of practitioners' fear-avoidance beliefs about physical activity and pain.

##### Practitioner behaviour

The outcome 'imaging referral excluding x-ray' has been included to estimate the effect of the intervention on practitioners' referral for other types of imaging. Including this outcome stems from a concern that while practitioners may reduce their use of x-rays, they may concurrently increase their use of other forms of imaging. Other forms of imaging are rarely recommended for acute LBP [11]. The outcomes 'advice to stay active' and 'advised bed rest' have been included as additional measures to assess the effectiveness of the intervention in changing practitioners' behaviour, with advice to stay active recommended, and advising bed rest not recommended. These outcomes are predicted to mediate the relationship between the intervention and patient LBP specific disability.

##### Predictors of practitioner behaviour

We have included measures of behavioural intention and other behavioural constructs (such as knowledge and beliefs about capabilities) as predictors of behaviour. Several outcomes measuring practitioners' intentions toward behaving in a manner consistent with the CPG's two recommendations are included because intention has been shown to be predictive of behaviour [58]. Intention is considered separately from the other behavioural constructs because it is thought to mediate the relationship between these constructs and actual behaviour. Details of the other behavioural constructs are reported in Additional File 2 - 'ALIGN outcome definitions' and Additional File 3 - 'ALIGN data collection instruments.'

##### Patient health outcomes

We have included pain severity as an additional measure of the effectiveness of the intervention on patient level health outcomes because pain severity is a recommended core outcome measure in LBP research [59]. In addition, it is useful to examine if the small benefits on pain observed in patient randomised trials, which underpinned the guidelines, are observed in a pragmatic setting with a less intensive patient level intervention.

We have included a preference-based measure of HRQoL in the set of patient-level outcomes to facilitate estimation of intervention effects in terms of quality adjusted life-years (QALYs). QALYs are commonly used in healthcare priority setting to compare interventions over multiple dimensions of HRQoL (in this case pain, pain-related disability, and physical function *inter alia*). Measurement of QALYs will permit the results of cost-effectiveness analyses to be expressed in terms of incremental cost per QALY.

##### Predictor of patient health behaviour

Fear-avoidance beliefs, measured at the patient level, will be included as a potential predictor of patient health behaviour. A recent systematic review investigating factors that were predictive of disability up to three months after the index consultation, identified a range of psychosocial factors, including depression, job physical demands, and FABs [60]. Fear-avoidance beliefs were more frequently shown to be associated with acute LBP compared with other psychosocial factors, and has been included for this reason.

##### Patient health behaviour

Patients will be asked if they received an x-ray. When patients do not receive either a referral for an x-ray or an x-ray from their practitioner, they may choose to see another practitioner who may provide an x-ray referral. While evidence of effectiveness of the intervention in changing x-ray referral rates by practitioners may be observed, it is of interest to examine if this ultimately changes the proportion of patients who receive an x-ray.

### Outcome measurement

Practitioner behaviour will be measured through practitioner-completed checklists of patient consultations and through clinical file audit. Practitioners will complete a checklist for each patient consultation over the two-week data collection period, indicating diagnostic procedures and interventions ordered, undertaken, or recommended. Outcomes measured through the checklists represent the practitioners' self-reported behaviour approximately three to four months post-symposium delivery. Clinical file audit will be undertaken for consenting patients. Outcomes measured through clinical file audit will represent practitioners' behaviour over the period zero to approximately seven months post-symposium delivery. The practitioner checklist and details of the data to be extracted from clinical file audit are available in Additional File 3.

Predictors of practitioner behaviour will be measured through a questionnaire, administered as a paper-based questionnaire or through the internet, at baseline and approximately four months following symposium delivery.

Patient health outcomes, patient health behaviour, and predictor of patient health behaviour, will be measured through a questionnaire, administered as a paper-based questionnaire or through the internet, at three months post-onset of their acute LBP episode. The patient questionnaire is available in Additional File 3.

Details of the outcome definitions are available in Additional File 2.

### Data quality assurance

Completed practitioner and patient questionnaires will be checked for errors and missing data as they are returned, and participants will be followed up to clarify anomalies. Double data entry will be used for paper-based questionnaires, and practitioner- and patient-completed checklists. When inconsistencies are found, these will be corrected by referring back to the paper-based version. Practitioner-completed checklists will be checked, as they are returned, to determine if there are items or sections that have consistent errors or missing data. When this occurs, practitioners will be followed up to discuss any misunderstandings in completing the checklists. Patient participants who have consented to follow-up data collection will be contacted if there is missing data on their patient completed checklist. Practitioners of these patient participants will be contacted if there is missing data on the practitioner-completed checklist. Processes will be put in place to monitor the number of practitioner- and patient-completed checklists received each day over the data collection period. A continuous sampling plan will be used to check a sample of data extracted from patient clinical files [61,62].

### Sample size

The primary practitioner outcome is x-ray referral. In our original sample size calculation for this outcome, we estimated that we would require 136 practices (68 physiotherapy and 68 chiropractic practices), each completing checklists for an average of 20 patient participants (providing a total of 2,720 patient participants), to provide 80% power to detect a difference of 10% in x-ray referral between intervention groups. This assumed a 39% x-ray referral rate in the control group, a 5% significance level, and an ICC of 0.10, and allowed for 20% attrition in practices. Empirical research has suggested ICCs of the order of 0.10 for process variables, such as x-ray referral, in primary care [63]. Data on x-ray referral rates were not available for the Australian context, so the x-ray referral rate of 39% was estimated from international research and assumptions regarding the process of x-ray referral in Australia. More specifically, for chiropractors, several international studies have estimated that referral for x-ray for acute LBP ranges from 62% to 72% [17,20,23]. For physiotherapists, we assumed that x-ray referral rates would be similar to those found in Australian general practice, which has been estimated at 28% [64], because many physiotherapists treat patients with LBP on referral from, and in conjunction with, GPs. Because we intended to recruit an equal number of patient participants from physiotherapy and chiropractic practices, we estimated the pooled x-ray referral rate to be 47%. Interventions comparing standard CPG dissemination with no intervention control groups have shown improvements in care of approximately 8% [4]. We therefore anticipated a decrease in the percentage of x-ray referral in the control group of this magnitude, providing an estimated referral rate of 39%.

As previously mentioned, we received a better than expected response from practices wishing to participate (133 physiotherapy and 77 chiropractic practices), and made a decision to include all practices because of uncertainties in the sample size calculation, including our estimate of ICC, the average number of patient participants for whom checklists are completed per practice (cluster size), variation in the cluster size, and the estimated x-ray referral rates. Assuming 168 practices complete the data collection (allowing for 20% attrition), we have investigated the likely width of the 95% CI for the risk difference and the log odds ratio for the primary practitioner outcome x-ray referral, assuming a range of values of the sample size parameters (Additional File 4 - 'ALIGN sample size calculations'). From this investigation, the width of the 95% CI for the observed difference in x-ray referral rates between groups is likely to be in the range of ±5% to ±7%. On the log odds scale, this is equivalent to a range of ±0.26 to ±0.41.

### Effectiveness analyses

#### Analysis subsets

The principle of intention-to-treat (ITT) is generally the recommended analysis approach for randomised trials because it maintains the comparability of the intervention groups, in known and unknown prognostic factors, brought about through randomisation, thus providing an unbiased estimate of intervention effect. In addition, non-compliance by practitioners and patients is allowed for, therefore providing an estimate of intervention effect that is more reflective of actual clinical practice [65,66]. For this reason, ITT analysis has been suggested for pragmatic trials [67].

In patient randomised trials, requirements of an ideal ITT analysis include compliance with the randomised intervention, no missing responses, and follow-up on all participants [65]. In a CRT, definition and application of an ITT analysis is more challenging [68] because of complexities with the hierarchical structure of the design and recruitment of participants potentially occurring post-randomisation of the clusters. More specifically, in a CRT such as ours, practices, practitioners, and patients may withdraw, or be lost to follow-up. In addition, during the recruitment phase of patient participants, practitioners may not actively recruit patients, potentially leading to empty clusters (this may be differential by group); they may recruit a differential number of participants depending on allocation status; or they may selectively recruit patients (leading to patients with different characteristics between groups) [68]. Statistical methods for handling missing data and dealing with selection bias in CRTs are limited [69], and for some forms of missing data (empty clusters), there is currently no statistical solution [68].

We therefore plan to present a modified ITT analysis as our primary analysis, where we will analyse clusters and participants (practitioners and patients) as they have been randomised, regardless of the intervention they have received, but will not impute missing data. As part of the secondary analyses, we plan to identify potential predictors of missing data through modelling, and include these predictors in the primary analysis model. Where possible, we plan to collect information on reasons for practice, practitioner, and patient withdrawal. To investigate potential selective recruitment, we will compare the practice list sizes [45], numbers of recruited participants, and their characteristics, between groups (examples of this include [70,71]).

#### Descriptive analyses at baseline

**Figure 2:**
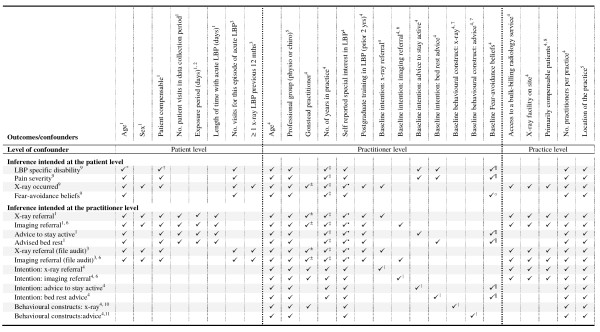
**Potential confounding variables adjusted for in the primary analyses**. ^1^Practitioner checklist; ^2^Exposure period is the number of data collection days post-patient entry into the trial. ^3^Clinical file audit; ^4^Practitioner questionnaire; ^5^Stratification variable; ^6^Imaging referral excluding x-ray; ^7 ^Adjusted for the baseline of the relevant behavioural construct (*e.g*., knowledge (Table 2)) for the specified behaviour (managing patients without referral for plain x-ray or advising patients to stay active); ^8^Practitioners answer yes to the question 'Do you primarily treat Work Cover (compensable) patients at your main practice?'; ^9^Patient questionnaire; ^10^Managing patients without referral for plain x-ray; ^11^Advising patients to stay active; *[100]; ^†^[100,101]; ^±^[102]; ^‡^[103]; ^·^[104]; ^II ^[76]; ^¶^[105]; °[106].

Descriptive statistics of demographic and potential confounding variables will be presented at baseline for the purposes of investigating the comparability of the intervention groups, and to provide descriptive information about the study sample. Potential confounding variables will include those presented in Figure 2, practitioners' self report of the average number of patients they treat per week, and the average number of acute LBP patients they treat per week (collected in the practitioner baseline questionnaire). In addition, we will compare between groups measures of the patients' pain (assessed by both the practitioner and patient) and general health (assessed by the practitioner) collected at the first visit of incident cases of acute LBP over the data collection period.

#### Primary analyses

We will estimate the effectiveness of the intervention on primary and secondary outcomes with marginal modelling using generalised estimating equations (GEEs). These models will appropriately account for correlation of responses within practice. We plan to fit an exchangeable correlation structure, where responses from the same practice are assumed to be equally correlated [50,72]. Additionally, we intend to use robust variance estimation that will provide valid standard errors even if the within-cluster correlation structure has been incorrectly specified [73,74]. For binary outcomes, a logit link function will be used. In the event that the ICC of an outcome for a particular analysis is negative, we will fit a generalised linear model with no adjustment for clustering (*e.g*., ordinary linear regression), making the assumption that in the context of cluster-based evaluations, negative ICCs are more likely to occur through sampling error than because of a true negative ICC [50].

Our primary analyses of outcomes will include adjustment for stratification variables (professional group and location of the practice) and pre-specified potential confounding variables (Figure 2). The potential confounders have been selected through discussion amongst the investigators and from published research (see Figure 2 notes). All pre-specified confounders will be included in the models even when no baseline imbalance exists. We have chosen this approach because confounder selection strategies that are based on collected data, (*e.g*., selecting confounders using preliminary statistical tests) result in models with poor statistical properties, such as incorrect type I error rates [75-78]. In the circumstance that there is limited data or events (*e.g*., advised bed rest), or both, to adjust for all confounders, we will report estimates of intervention effect from models including only the stratification variables, and baseline of the outcome variables where appropriate (FAB, intention, and behavioural constructs).

Estimates of intervention effect from these models with binary outcomes will yield odds ratios. In addition to the odds ratios, we plan to also provide estimates of risk difference to aid interpretability and provide greater information to fully assess the implications of the intervention effects [79]. Estimates of risk difference will be estimated from GEEs, with robust variance estimation, using an identity link function [80]. For each outcome, the estimate of intervention effect and its 95% CI will be provided. For the primary outcomes, we plan to provide estimates of ICCs and their 95% CIs.

Regression diagnostics will be used to assess the influence of outliers on estimates of intervention effect and for analysing residuals. No adjustment will be made for multiple testing. All tests will be two-sided and carried out at the 5% level of significance.

#### Secondary analyses

We will conduct a subgroup analysis to examine whether the effect of the intervention on x-ray referral is modified by professional group (physiotherapist versus chiropractor). We hypothesise that the effect of the intervention may differ by professional group because x-rays are seen as more integral to the treatment of patients for chiropractors compared with physiotherapists [22]. We will examine this by fitting a model that includes an interaction term between intervention group and professional group. The estimated ratio of odds ratios and its 95% CI will be presented from this model.

GEEs will yield unbiased estimates of intervention effect only when data are missing completely at random, or when known covariates that are associated with the missing data mechanism are included in the model (missing at random) [69]. As part of the secondary analyses, we plan to identify potential predictors of missing data through modelling (*e.g*., [81]), and include these predictors in the primary analysis model. We will investigate methods to impute missing outcome data collected at baseline (*e.g*., baseline behavioural constructs, FAB) [82]. Finally, we will investigate methods to adjust for potential differential recruitment [68].

A disadvantage of using GEEs is that only one level of clustering can be modelled. For data collected at the patient level, there are three levels of data: practices, practitioners, and patients. It is possible to allow for clustering at the practice level or the practitioner level. While we have chosen to adjust for clustering at the practice level, because this is the unit of randomisation, there may be an argument for adjusting at the practitioner level if the variation at this level is larger; this could be predicted to arise because the practitioner has a more direct influence on patient outcomes (such as x-ray referral). We will therefore undertake sensitivity analyses investigating the impact of allowing for clustering at the level of the practitioner.

We plan to undertake explanatory analyses to investigate if intervention effects are mediated by our hypothesised predictors of practitioner behaviour [83-85]. Detailed description of our theoretical model and results of these explanatory analyses will form separate publications.

Finally, we plan to undertake analyses comparing the outcomes (*e.g*., 'x-ray referral,' 'imaging referral excluding x-ray') measured via different methods of data collection (practitioner completed checklists, patient completed checklists, file audit). In addition, we will undertake statistical analyses comparing proxy (*e.g*., clinical decisions in response to vignettes) and direct methods of measuring clinical behaviour [86]. These analyses will be undertaken to add to our knowledge about the design of future trials, but will not be considered part of the trial effectiveness analyses, and will inform a separate publication.

### Economic evaluation

Active implementation entails an additional cost that may or may not be offset by health gains and/or reductions in health service utilisation. Evidence is limited regarding this trade-off between incremental costs (savings) and effects for active implementation versus simple dissemination of practice guidelines for management of LBP. Hoeijenbos *et al*. [87] conducted a cost-utility analysis comparing standard dissemination versus active implementation of the Royal Dutch Physiotherapy Association (RDPA) guideline for non-specific LBP based on data from the Bekkering CRT [40,41]. While lower per patient healthcare utilisation in the active implementation group (mean = EUR 125, SD = EUR 91 versus mean = EUR 145, SD = EUR 95, p = 0.026) offset the relatively modest incremental treatment cost of active implementation (EUR 366 per physiotherapist), the observed differences in healthcare utilisation (and health outcomes) could not be attributed to an intervention effect due to failure to control for pre-existing between-group differences in patient characteristics. Hoeijenbos *et al*. [87] therefore concluded that 'it is very likely that the extended implementation strategy incurs extra costs without producing health gains, hence it is very likely to be not cost-effective.'

The implementation strategy employed by Bekkering *et al*. [40,41] and the RDPA guideline differ from the ALIGN intervention and the NHMRC guideline. Importantly, the RDPA guideline contains no specific recommendation that would limit referral for imaging [88], and the implementation strategy employed by Bekkering *et al*. [40,41] made no attempt to target referral behaviour for imaging. Another recently completed study [33,89] will shortly report on the cost-effectiveness of active implementation versus standard dissemination of the NHMRC guideline, but its findings will relate to general practice rather than to the allied health setting. Findings from the economic evaluation described here will therefore complement rather than replicate previous findings.

Economic evaluation alongside the ALIGN CRT will be conducted with the aim of quantifying additional costs (savings) and health gains arising from the ALIGN intervention as compared to access to the guideline via existing practice. Secondary aims will be to determine whether the incremental treatment costs of the ALIGN intervention are offset by reductions in health service expenditure (*i.e*., whether implementing the guideline is cost-saving as compared with existing practice), and to determine whether the ALIGN intervention dominates existing practice (*i.e*., less costly but no less effective). The time horizon for inclusion of relevant costs and consequences for the trial-based evaluation described here coincides with the final scheduled follow-up of patient participants (three months post-onset of their acute LBP episode).

The proposed economic evaluation will take a societal perspective in identifying, measuring, and valuing costs and consequences, but some cost categories likely to produce clinically and economically insignificant variation in incremental cost will be excluded to simplify our analysis [90]. Likewise, we exclude some dimensions of health and HRQoL not captured by the patient-level outcomes and some (but not all) non-health outcomes (*e.g*., social status) on the grounds that their inclusion would be unlikely to be alter policy recommendations. Research and evaluation costs will be excluded except where they might plausibly contribute to a clinically significant treatment effect. Costs common and invariant to both intervention and control groups (*e.g*., costs associated with development and standard dissemination of the guideline) will not be explicitly calculated for the incremental analysis described here.

#### Identification, measurement, and valuation of health outcomes

In line with the main analysis, the primary outcome for the economic analysis will be x-ray referral to three months post-onset of patients' acute LBP episode based on the practitioner checklist. For the economic analysis, we will conduct a secondary analysis under the assumption that referral implies occurrence such that patients will be coded as 'x-ray received' if data from any one of the practitioner completed checklist, clinical file audit, or patient questionnaire indicate x-ray referral or occurrence. Patients will be coded as 'x-ray avoided' if x-ray referral or occurrence is nowhere indicated in data from the practitioner completed checklist, clinical file audit, and patient questionnaire. For the purposes of the economic analysis, x-ray referral and x-ray avoided are intermediate measures of mortality and morbidity effects that occur beyond trial-end arising due to reduced exposure to ionising radiation [91]. Intervention effects with respect to x-ray referral and x-ray avoided to three months post-onset will be estimated using methods specified for the main analysis, controlling for the set of potential confounders specified above.

Additional secondary outcomes for the economic analysis are: any imaging referral (including lumbosacral plain x-ray, full spine plain x-ray, lumbar CT scan, lumbar MRI, or bone scan) and any imaging avoided to three months post-onset, LBP-specific disability at three months post-onset, and HRQoL at three months post-onset. The rationale for measuring referral/avoidance of any imaging in the economic analysis is as described above for the outcome 'imaging referral excluding x-ray.' Intervention effects with respect to any imaging referral/avoidance to three months post-onset will be estimated using methods specified for the main analysis, controlling for the set of potential confounders specified above. LBP-specific disability and HRQoL are included to provide measures of differential health outcomes arising within the trial period due to *inter alia *any between-group difference in providing advice to stay active. Intervention effects with respect to LBP-specific disability will be taken from the main analysis of Roland-Morris Disability Questionnaire (RDQ) at three months post-onset of their acute LBP episode, controlling for the set of potential confounders specified above.

Preference-based HRQoL weights derived from the patient-level AQoL-4D (Assessment of Quality of Life) responses must be combined with a period of time to permit estimation of intervention effects in QALY terms (and incremental cost-effectiveness in cost per QALY terms). Note, however, that follow up of LBP specific disability and HRQoL for the ALIGN study is scheduled for a single time point at three months post-onset. Due to feasibility/resource constraints and to minimise patient attrition at the primary endpoint, we decided not to schedule repeated observations on patient level outcome measures in the present trial. Findings from two studies that informed development of the guideline [92,93] suggest that any intervention effect with respect to pain is likely to arise in the intermediate term (3 to 12 weeks) rather than in the short term (<3 weeks). Based on this limited information as to the time-path of any intervention effect, patients are assumed to transition from their baseline health state to their observed health state (as measured by HRQoL at three months post-onset) at three weeks post-onset, remain stable in their observed health state (as measured by HRQoL at three months post-onset) during the nine weeks from three to twelve weeks post-onset, before transitioning to full or normal health by trial-end. Intervention effects with respect to total QALYs are therefore estimated as the difference between curves for treatment and control groups over the nine weeks from three to twelve weeks post-onset of their acute LBP episode using methods specified for the main analysis of patient level clinical outcome measures and controlling for the set of potential confounders specified above. No attempt has been made to extrapolate from any between-group difference at trial-end based, for example, on secondary data as to the relative stability of pain, disability, and return to work between three and twelve months post-onset [94]. Any between-group differences in transition to chronic lower back pain are therefore excluded from our estimates of incremental effectiveness.

#### Identification, measurement, and valuation of resource use

Incremental costs will reflect resource use associated with development of the implementation intervention, delivery of the implementation strategy, and any changes in clinical practice and subsequent health effects. Methods for the ALIGN cost-analysis will be as described by Mortimer *et al*. [89] for the IMPLEMENT trial (planned analyses) but paid and unpaid costs of homecare will be excluded on the grounds that any between-group variation in homecare costs is expected to be quantitatively unimportant.

Given the characteristic distribution of health costs (truncated at zero and right skewed), the importance of obtaining readily interpretable marginal effects, and our interest in population-average effects, we will model intervention effects on health service expenditure and total costs using one-part GEEs with a log link rather than transformed ordinary least squares or two-part models [95]. Specification of the log link for the GEE model permits natural interpretation of marginal effects on cost without retransformation [95]. Correlation structure, standard errors, and controls for confounding variables will be as specified for the main analysis.

#### Adjustment for differential timing

All costs will be inflated to current AUD for the year of study completion. All costs and benefits will be converted to present values for the year of study completion using an annual discount rate of 5% in the base-case, and annual rates of 3% and 7% in sensitivity analysis.

#### Incremental analysis

Results from the economic evaluation will be expressed as: additional costs (savings) per x-ray referral and per x-ray avoided to three months post-onset; additional costs (savings) per any imaging referral and per any imaging avoided to three months post-onset; additional costs (savings) per point difference in RDQ at three months post-onset; and additional costs (savings) per QALY gained.

#### Uncertainty

Cost-effectiveness acceptability curves (CEACs) will be derived from the joint density of incremental costs (ΔC) and incremental effects (ΔE) for the intervention as compared to existing practice. The joint density will be obtained via non-parametric bootstrapping from the distribution of observed cost/effect pairs for patient participants. Separate CEACs will be derived for upper/lower bound estimates of a pre-specified set of uncertain parameters not estimated with sampling error: development costs (none, full); unit cost for practitioner participant time (AUD 0, average hourly wage rate), unit cost for patient travel/waiting time to attend treatment (AUD 0, average hourly wage rate), and the discount rate (3%, 7%).

### Publication policy

The investigators of the grant will be responsible for ensuring the results of the trial are published within a reasonable timeframe after conclusion of the trial. The results from the trial will be published regardless of the outcome. Reporting of this trial will adhere to the relevant, and most up-to-date, CONSORT (Consolidated Standards of Reporting Trials) statements at the time of submission [79,96-99].

### Ethical review

Ethical approval for this trial was obtained from the Monash University Human Research Ethics Committee (CF07/1060; CF09/1956). The investigators will ensure the trial is conducted in compliance with this protocol and the Australian National Statement on Ethical Conduct in Human Research [52].

## Competing interests

SEG, JMG, and SM are members of the editorial board of Implementation Science. DAO is an associate editor of Implementation Science. JEM, DAO, SDF, and SEG have received research funding for a different study from the Physiotherapists Registration Board of Victoria. SDF has received research funding from the Chiropractors Registration Board of Victoria and from the Chiropractors Association of Australia. SEG and JLK have previously practised as physiotherapists, and SDF and BFW have previously practised as chiropractors, from which they have all derived an income. JEM and SEG are funded to provide methods advice to guideline developers through Australia's NHMRC. The remaining authors declare that they have no competing interests.

## Authors' contributions

SEG, JEM, JMG, DSM, JLK, BFW, DAO, and SDF conceptualised and designed the study, and secured funding; SEG was the lead investigator of the funding application. MJP provided input on the design. DAO, SM, JJF, SEG, MJP, JLK, BFW, SDF, and JMG designed the intervention. JEM wrote the first draft of this publication excluding the background, description of the interventions and intervention fidelity, and the economic evaluation. The former two were contributed by MJP and the latter by DSM. All authors contributed to revisions of the manuscript and take public responsibility for its content.

## Supplementary Material

Additional file 1 **ALIGN intervention content**. This file includes an outline of the symposium day, including a brief description of the content in each session. Click here for file

Additional file 2 **ALIGN outcome definitions**. This file provides outcome definitions, including details of the measurement instruments used. Click here for file

Additional file 3 **ALIGN data collection instruments**. This file includes practitioner and patient data collection instruments including the practitioner vignettes, details of the measurement instruments for the behavioural constructs, practitioner checklist (for physiotherapists), patient checklist, questionnaire for patients, fear-avoidance beliefs questionnaire modifications, and data to be extracted from clinical file audit. Click here for file

Additional file 4 **ALIGN sample size calculations**. This file includes details of the assumptions and calculations in estimating the likely width of the 95% CI for the risk difference and the log odds ratio, for the primary practitioner outcome, x-ray referral. Click here for file
