# Supplementary material for: Improving the care for people with acute low-back pain by allied health professionals (the ALIGN trial): A cluster randomised trial protocol
Source: Implement Sci. 2010 Nov 10;5:86. doi: 10.1186/1748-5908-5-86 (PMC2994785; doi:10.1186/1748-5908-5-86)
Supplement: Additional file 1 — ALIGN intervention content. This file includes an outline of the symposium day, including a brief description of the content in each session. [file 1748-5908-5-86-S1.PDF]

ALIGN CRT Intervention Content

The intervention for the ALIGN CRT consisted of a full-day weekend symposium-style event (with program and written material supporting presentations/content covered). The first half of the symposium focused on awareness raising and address the following theoretical domains: beliefs about professional role, social influences, beliefs about consequences and knowledge. The second half of symposium included more small group/interactive activity to address beliefs about capabilities and to allow for skill development regarding both target behaviours.

A summary of the content delivered in each session of the symposium is outline below.

Time	Content
9:00am	*Welcome and introductions (by study investigator)*
9:20am	*Audience straw poll #1 (lead by study investigator)*<br>Using audience response system software, 10 questions measuring knowledge, attitudes, beliefs and intentions about acute low-back pain management were posed to the audience, who could respond anonymously using wireless keypads, and aggregated results of participants were presented in real time
9:35am	*Keynote speech by peer opinion leader*<br>Speech given by high status person recognised within the profession to discuss professional standards, state-of-the-art in diagnosis and/or communicating with patients with acute low-back pain, including use of latest research evidence and clinical practice guidelines
10:20am	*Video recording by peer opinion leader*<br>Pre-recorded video of well respected clinician conveying confidence in diagnosis without plain x-ray, dispelling fears around missed pathology and/or litigation, and conveying importance of reassuring patients
10:30am	*Video recording by radiologist*<br>Pre-recorded video of radiologist outlining the amount of radiation delivered by plain x-ray and its poor utility in acute uncomplicated low-back pain
10:35am	*Video recording by consumer advocate*<br>Pre-recorded video or consumer advocate describing their expectation of a good health professional (those who are good listeners/ communicators, provide good explanations/ reassurance, and provide evidence-informed best care)
10:40am	Morning tea
11:00am	*Small group discussion*<br>Discussion between six participants lead by a clinician table facilitator , to discuss how the participants currently manage people with acute low-back pain, what participants think of the guideline and its recommendations, any scenarios participants find managing patients in a manner consistent with the guideline (i.e., less plain x-rays, giving advice to stay active) difficult, and strategies to overcome these
12:00pm	*Skills demonstration (managing acute LBP patients without x-ray)*<br>Demonstration / modelling of skills needed to diagnose and manage patients without plain x-ray by an experienced clinician
12:15pm	*Skills demonstration (advising acute LBP patients to stay active)*<br>Demonstration / modelling of relevant skills in a successful communication encounter (giving advice to stay active) with patient (e.g. strategies on how to

	develop rapport, convey empathy, confidence, reassure patient, give message that patient will improve over time etc) by experienced clinician
12:40pm	Lunch
1:45pm	*Small group practical: Simulated patients*<br>Rehearsal of diagnostic and communication skills on trained simulated patients (x4) in groups of six participants, lead by a clinician table facilitator
3:00pm	Afternoon tea
3:15pm	*Reflection lead by peer opinion leader*<br>Brief summary/reflection of the key messages presented throughout the day, with opportunity for questions from the audience
3:45pm	*Audience straw poll #2 (lead by study investigator)*<br>Using audience response system software, 3 questions measuring beliefs about the extent to which participants believe their management of acute low-back pain patients will change were posed to the audience, who could respond anonymously using wireless keypads, and aggregated results of participants were presented in real time
4:00pm	*Summary and evaluation*
4:30pm	Close

Two-four weeks following the symposium, participants received a follow-up telephone call by a clinician member of project team to enquire about any difficulties encountered in implementing behaviours (and strategies to overcome these) and to discuss how practice has changed (academic detailing style).
