# Supplementary material for: Improving the care for people with acute low-back pain by allied health professionals (the ALIGN trial): A cluster randomised trial protocol
Source: Implement Sci. 2010 Nov 10;5:86. doi: 10.1186/1748-5908-5-86 (PMC2994785; doi:10.1186/1748-5908-5-86)
Supplement: Additional file 2 — ALIGN outcome definitions. This file provides outcome definitions, including details of the measurement instruments used. [file 1748-5908-5-86-S2.PDF]

ALIGN outcome definitions

In this additional file we provide details of how each outcome, listed in Table 1 of the ALIGN protocol, will be defined, including information on the measurement instruments used. A full list of abbreviations used in the following definitions are available in the ALIGN protocol.

X-ray referral

The dichotomous variable ‘x-ray referral’, will be coded as ‘Yes’ if the practitioner ticks either “Lumbosacral plain x-ray” or “Full spine plain x-ray” at any of the patient consultations over the two week data collection period. Absence of these selections will be coded ‘No’.

Advice to stay active

The dichotomous variable ‘advice to stay active’, will be coded as ‘Yes’ if the practitioner ticks “Advice to stay active” at any of the patient consultations over the two week data collection period, regardless of other interventions selected. Absence of this selection will be coded ‘No’.

Imaging referral excluding x-ray

The dichotomous variable ‘imaging referral excluding x-ray’, will be coded as ‘Yes’ if the practitioner ticks “Lumbar CT scan”, “Lumbar MRI”, or “Bone scan”, at any of the patient consultations over the two week data collection period. Absence of these selections will be coded ‘No’.

Advised bed rest

The dichotomous variable ‘advised bed rest’, will be coded ‘Yes’ if the practitioner indicates “Bed rest” for greater than two days at any of the patient consultations over the two week data collection period. Absence of this indication will be coded ‘No’.

X-ray referral (file audit)

The dichotomous variable ‘x-ray referral (file audit)’, will be coded ‘Yes’ if there is evidence from clinical file audit of either lumbosacral plain x-ray or full spine plain x-ray over the three month period post onset of acute low-back pain (LBP). Evidence of referral includes any of the following: referral letter to a general practitioner, referral noted, copy of media report, report, findings noted, or other evidence of either referral or imaging. Absence of this evidence will be coded ‘No’.

Imaging referral excluding x-ray (file audit)

The dichotomous variable ‘imaging referral excluding x-ray (file audit)’, will be coded ‘Yes’ if there is evidence from clinical file audit of lumbar CT scan, lumbar MRI, or bone scan, over the three month period post onset of acute LBP. Evidence of referral includes any of the following: referral letter to a general practitioner, referral noted, copy of media report, report, findings noted, or other evidence of either referral or imaging. Absence of this evidence will be coded ‘No’.

Intention to adhere to CPG recommendations

Intention to behave in a manner consistent with the guideline recommendations for x-ray referral, imaging referral excluding x-ray, advice to stay active, and bed rest advice, will be measured through four vignettes representing hypothetical scenarios

about patients presenting with acute LBP (available in Additional file 3 – "ALIGN data collection instruments"). The four vignettes, developed for the IMPLEMENT trial [1], differ in factors that have been identified [2-5] as being potentially predictive of GPs' intention to practice in a manner consistent with guideline recommendations [6]. For each vignette, practitioners will indicate on a checklist the diagnostic procedures and interventions they would order, undertake, or recommend. This proxy method of measuring practitioner behaviour is also referred to as 'simulated behaviour' or 'simulated case' in similar studies of health practitioner behaviour [3, 7]. The checklist options are the same as those provided on the practitioner checklists for each patient encounter. Dichotomous outcomes of intention to adhere to the CPG recommendations for the four behaviours will be created as per the descriptions above for the outcomes 'x-ray referral', 'advice to stay active', 'imaging referral excluding x-ray', and 'advised bed rest'. 'X-ray referral', 'imaging referral excluding x-ray', and 'advised bed rest', will be reverse coded to reflect intention to adhere to the CPG recommendations.

Additional measures of behavioural intention will be included for the behaviours managing patients without referral for x-ray and advising patients to stay active. Generalised behavioural intention measures the practitioner's strength of intention to perform a particular behaviour, while behavioural intention performance measures how often the practitioner intends to perform the behaviour. Details of the number of items for each measure of intention, examples of the items, and scoring, are available in Additional file 3.

Other behavioural constructs

Behavioural constructs, in addition to intention, will be measured for the behaviours managing patients without referral for x-ray and advising patients to stay active. These include constructs within the following domains: beliefs about capabilities, beliefs about consequences, knowledge, professional role and identity, social influences, environmental context and resources, and memory. Definitions of the construct domains are available in Table 2 of the ALIGN protocol. The questionnaire measuring the constructs was developed following qualitative analysis of semi-structured interviews with physiotherapists and chiropractors and represents a modification of a survey instrument previously used and tested for validity and reliability in a general practice population [8]. Details of the number of items for each behavioural construct, examples of the items, and scoring, are available in Additional file 3. The questionnaire is available on request.

We have included an additional measure of the construct domain beliefs about consequences, based on previous research which has suggested that practitioners' fear-avoidance beliefs can influence their adherence to LBP guidelines [9, 10] and that practitioners' beliefs about back pain can influence their treatment recommendations [11, 12].

LBP specific disability

Low-back pain specific disability will be measured using the Roland-Morris Disability Questionnaire (RDQ) [13]. The RDQ includes 24 statements of limitations in aspects of daily living that patients may experience due to LBP. A disability score for each participant is created from the number of limitations they select, ranging

from 0 to 24. Reliability and validity for the questionnaire is reported in Roland et al [13, 14].

Pain severity

Pain severity (intensity) will be measured using a modified version of the characteristic pain intensity subscale of the Graded Chronic Pain Scale [15]. The characteristic pain intensity subscale consists of three items measuring current, worst, and average pain, over a specified period. We plan to use these items, and an additional item measuring least pain, over the previous week [16, 17]. Each item will be measured on an eleven point numerical rating scale; 0 indicating no pain and 10 indicating pain as bad as it could be. An average of the scores for the four items will then be calculated, providing an overall pain severity score ranging from 0 – 10.

X-ray occurred

The dichotomous variable ‘x-ray occurred’, will be coded according to the patient participant’s response to the question “Have you received an x-ray for this episode/flare-up of your back pain?”.

Fear-avoidance beliefs

Fear-avoidance beliefs at the patient participant level will be measured using the Fear-Avoidance Beliefs-Questionnaire physical activity subscale [18]. This consists of four items, each measured on a seven point Likert scale from completely disagree to completely agree (1 – 7; converted to 0 – 6 for the purpose of scoring). The scores are summed across the four items to create a fear-avoidance score for each patient participant, ranging from 0 to 24, with higher scores reflecting greater fear-avoidance. Details of reliability, validity, and responsiveness are available in Waddell et al [18] and George et al [19].

Health-related quality of life

Health-related quality of life (HRQoL) will be measured using the four dimension Assessment of Quality of Life (AQoL-4D) instrument [20]. The AQoL-4D can be used as a multi-attribute utility instrument, yielding a preference-based measure of HRQoL for use in calculating quality adjusted life-years (QALYs). The AQoL-4D’s four dimensions include independent living (self-care, household tasks, and mobility), social relationships (relationships with others, social isolation and family role), physical senses (seeing, hearing and communication), and psychological well-being (sleep, anxiety and depression). These dimensions encompass LBP-related variation in pain, pain-related disability and physical function. The validity and reliability of the AQoL-4D for the measurement of preference-based HRQoL has been demonstrated in the Australian general population [21]. The AQoL-4D for self-completion is reproduced in Additional file 3.

References

1. McKenzie JE, French SD, O'Connor DA, Grimshaw JM, Mortimer D, Michie S, Francis J, Spike N, Schattner P, Kent PM, Buchbinder R, Green SE: **IMPLEmenting a clinical practice guideline for acute low back pain evidence-based manageMENT in general practice (IMPLEMENT): Cluster randomised controlled trial study protocol.** *Implement Sci* 2008, **3:**11.

2. Australian Acute Musculoskeletal Pain Guidelines Group (AAMPGG): **Evidence-based management of acute musculoskeletal pain (http://www.nhmrc.gov.au/publications/synopses/cp94syn.htm). Australian Academic Press: Brisbane.** 2003.
3. Bonetti D, Eccles M, Johnston M, Steen N, Grimshaw J, Baker R, Walker A, Pitts N: **Guiding the design and selection of interventions to influence the implementation of evidence-based practice: an experimental simulation of a complex intervention trial.** *Soc Sci Med* 2005, **60:**2135-2147.
4. Buchbinder R, Jolley D, Wyatt M: **2001 Volvo Award Winner in Clinical Studies: Effects of a media campaign on back pain beliefs and its potential influence on management of low back pain in general practice.** *Spine (Phila Pa 1976)* 2001, **26:**2535-2542.
5. O'Connor D, Green S, French S, Grimshaw J, Spike N, Schattner P, King S, Michie S, Francis J, McKenzie J: **Using a theoretical framework to identify barriers and enablers to the uptake of an evidence-based clinical practice guideline in general practice.** In *Health for All? General Practice and Primary Health Care Research Conference*. Hobart, Australia; 2008.
6. French SD: **Developing and testing complex interventions for improving the use of evidence in clinical practice: a case study of the management of acute low-back pain in general practice.** Monash University, Monash Institute of Health Services Research; 2008.
7. Hrisos S, Eccles MP, Francis JJ, Dickinson HO, Kaner EF, Beyer F, Johnston M: **Are there valid proxy measures of clinical behaviour? a systematic review.** *Implement Sci* 2009, **4:**37.
8. O’Connor D, Green S, French S, McKenzie J, King S, Francis J, Grimshaw J, Michie S, Spike N, Schattner P, and the IMPLEMENT Study Group: **Identifying general practitioner beliefs, intentions and behaviour toward uptake of an evidence-based guideline for acute low-back pain using the theory of planned behaviour.** In *4th Guidelines International Network (G-I-N) Conference*; *22-25 August 2007; Toronto, Canada*. 2007
9. Coudeyre E, Rannou F, Tubach F, Baron G, Coriat F, Brin S, Revel M, Poiraudeau S: **General practitioners' fear-avoidance beliefs influence their management of patients with low back pain.** *Pain* 2006, **124:**330-337.
10. Linton SJ, Vlaeyen J, Ostelo R: **The back pain beliefs of health care providers: are we fear-avoidant?** *J Occup Rehabil* 2002, **12:**223-232.
11. Houben RM, Gijsen A, Peterson J, de Jong PJ, Vlaeyen JW: **Do health care providers' attitudes towards back pain predict their treatment recommendations? Differential predictive validity of implicit and explicit attitude measures.** *Pain* 2005, **114:**491-498.
12. Houben RM, Ostelo RW, Vlaeyen JW, Wolters PM, Peters M, Stomp-van den Berg SG: **Health care providers' orientations towards common low back pain predict perceived harmfulness of physical activities and recommendations regarding return to normal activity.** *Eur J Pain* 2005, **9:**173-183.
13. Roland M, Morris R: **A study of the natural history of back pain. Part I: development of a reliable and sensitive measure of disability in low-back pain.** *Spine (Phila Pa 1976)* 1983, **8:**141-144.
14. Roland M, Fairbank J: **The Roland-Morris Disability Questionnaire and the Oswestry Disability Questionnaire.** *Spine* 2000, **25:**3115-3124.

15. Von Korff M, Ormel J, Keefe FJ, Dworkin SF: **Grading the severity of chronic pain.** *Pain* 1992, **50:**133-149.
16. Hägg O, Fritzell P, Nordwall A: **The clinical importance of changes in outcome scores after treatment for chronic low back pain.** *Eur Spine J* 2003, **12:**12-20.
17. Mannion AF, Balagué F, Pellisé F, Cedraschi C: **Pain measurement in patients with low back pain.** *Nat Clin Pract Rheum* 2007, **3:**610-618.
18. Waddell G, Newton M, Henderson I, Somerville D, Main CJ: **A Fear-Avoidance Beliefs Questionnaire (FABQ) and the role of fear-avoidance beliefs in chronic low back pain and disability.** *Pain* 1993, **52:**157-168.
19. George SZ, Fritz JM, McNeil DW: **Fear-avoidance beliefs as measured by the fear-avoidance beliefs questionnaire: change in fear-avoidance beliefs questionnaire is predictive of change in self-report of disability and pain intensity for patients with acute low back pain.** *Clin J Pain* 2006, **22:**197-203.
20. Hawthorne G, Richardson J, Day N: *Technical Report 12: Using the Assessment of Quality of Life (AQoL) Version 1.* Melbourne: Centre for Health Program Evaluation, Monash University; 2000.
21. Hawthorne G, Richardson J, Day NA: **A comparison of the Assessment of Quality of Life (AQoL) with four other generic utility instruments.** *Ann Med* 2001, **33:**358-370.
