# Supplementary material for: Improving the care for people with acute low-back pain by allied health professionals (the ALIGN trial): A cluster randomised trial protocol
Source: Implement Sci. 2010 Nov 10;5:86. doi: 10.1186/1748-5908-5-86 (PMC2994785; doi:10.1186/1748-5908-5-86)
Supplement: Additional file 3 — ALIGN data collection instruments. This file includes practitioner and patient data collection instruments including the practitioner vignettes, details of the measurement instruments for the behavioural constructs, practitioner checklist (for physiotherapists), patient checklist, questionnaire for patients, fear-avoidance beliefs questionnaire modifications, and data to be extracted from clinical file audit. [file 1748-5908-5-86-S3.PDF]

ALIGN Data Collection Instruments

Contents

VIGNETTES FOR PRACTITIONERS……………………………………………………………………….....2

DETAILS OF THE OUTCOME MEASUREMENT FOR THE BEHAVIOURAL CONSTRUCTS………..…7

PRACTITIONER CHECKLIST (FOR PHYSIOTHERAPISTS)……………………….……………………10

PATIENT CHECKLIST……………………………………………..…………………………………..…12

QUESTIONNAIRE FOR PATIENTS………………..…………………………………………………..…14

FEAR AVOIDANCE BELIEFS QUESTIONNAIRE (FAB-Q) MODIFICATIONS…………………….…...22

CONTENT FOR COLLECTION IN FILE AUDIT…………………………………………………….….…...23

VIGNETTES FOR PRACTITIONERS

PART A

This section contains five hypothetical scenarios about patients who present to you with acute low-back pain. In the scenarios we have varied a range of features that might influence your management decisions (in regards to investigations you might order and interventions you might recommend or undertake). At the end of each scenario we ask you to indicate what investigation(s) you would order for the patient described in the scenario, and what intervention(s) you would recommend or undertake for this patient. We are aware that the scenario format means that skills you may normally draw on, such as evaluating non-verbal cues from the patient and performing a physical examination, cannot be a factor in your assessment. Nevertheless, given this understanding, we hope that you address each scenario and answer the questions as best as you can with the information provided. We have left space for you to comment on your decisions, if you wish.

Scenario 1

A 48 year old office worker attends your clinic. He is usually very active, playing lots of sport and doing regular exercise (e.g. jogging, gym). He has low-back pain, rated 5 out of 10. The pain started two weeks ago and is located in the low-back region, right sided, no radiation. The pain is relieved by stretching his low-back and using a heated wheat bag. The pain is worse after playing sport, to the point where in the last week he had to stop mid-game during basketball. He has no previous history of low-back pain. The patient thinks that an x-ray is required to "find out what is wrong", and he is fearful that movement and activity might make the pain worse.

1. Which investigations would you order, undertake or recommend for this patient at this visit? *Please tick all that apply:*

☐ Lumbosacral plain x-ray
☐ Lumbar CT scan
☐ Lumbar MRI
☐ Bone scan
☐ Full spine plain x-ray
☐ Other 1 (*please specify*) ___________________________
☐ Other 2 (*please specify*) ___________________________
☐ Other 3 (*please specify*) ___________________________
☐ None

2. Which interventions would you recommend or undertake for this patient at this visit? *Please tick all that apply:*

❑ Spinal manipulation
❑ Spinal adjustment (please specify) ___________________________
❑ Mobilisation
❑ Other techniques (please circle or specify): Activator / SOT blocks / Flexion distraction / Other (specify) _________________________
❑ Massage
❑ Acupuncture / dry needling
❑ Electrotherapy (e.g. TENS, interferential, ultrasound)
❑ Thermal modalities (e.g. heat, ice)
❑ Spinal traction
❑ Bed rest for ____ days (please specify number of days)
❑ Advice to stay active
❑ Advice regarding alternate ways of moving or performing activities
❑ Advice to avoid pain provoking movements
❑ Paracetamol
❑ Non-steroidal anti-inflammatory drugs (NSAIDs)
❑ Back exercises
❑ General exercise (e.g. walking)
❑ Lumbar supports
❑ Printed information (please specify) ___________________________
❑ Work modification
❑ Other 1 (please specify) ___________________________
❑ Other 2 (please specify) ___________________________
❑ Other 3 (please specify) ___________________________
❑ Referral to another health care provider (e.g. another chiropractor or physiotherapist, general practitioner, pain clinic, specialist).
If yes, please specify ___________________________

If you wish to comment on your management decisions, please do so here:

____

____

____

____

____

Scenario 2

A 57 year old office worker sees you for low-back pain. She says her pain began 8 weeks ago. There was no specific incident that caused the pain. The pain is located in the lower back region, with no radiation. The pain is a dull ache (3 out of 10), with occasional sharp "twinges" with certain movements. The pain is relieved by heat and a massage from her spouse. She has no history of low-back pain. The patient is overweight (BMI 30), has mild hypertension, and a family history of type 2 diabetes. The patient rarely does any exercise. During the consultation she indicates to you that she is anxious that she may have a serious disease. The patient says "a friend had low-back pain like this and they had an x-ray and it showed that they really had something major wrong with them". She repeatedly requests an x-ray during the consultation.

1. Which investigations would you order, undertake or recommend for this patient at this visit? *Please tick all that apply:*

☐ Lumbosacral plain x-ray
☐ Lumbar CT scan
☐ Lumbar MRI
☐ Bone scan
☐ Full spine plain x-ray
☐ Other 1 (*please specify*) ___________________________
☐ Other 2 (*please specify*) ___________________________
☐ Other 3 (*please specify*) ___________________________
☐ None

2. Which interventions would you recommend or undertake for this patient at this visit? *Please tick all that apply:*

❑ Spinal manipulation
❑ Spinal adjustment (please specify) ___________________________
❑ Mobilisation
❑ Other techniques (please circle or specify): Activator / SOT blocks / Flexion distraction / Other (specify) __________________________
❑ Massage
❑ Acupuncture / dry needling
❑ Electrotherapy (e.g. TENS, interferential, ultrasound)
❑ Thermal modalities (e.g. heat, ice)
❑ Spinal traction
❑ Bed rest for ____ days (please specify number of days)
❑ Advice to stay active
❑ Advice regarding alternate ways of moving or performing activities
❑ Advice to avoid pain provoking movements
❑ Paracetamol
❑ Non-steroidal anti-inflammatory drugs (NSAIDs)
❑ Back exercises
❑ General exercise (e.g. walking)
❑ Lumbar supports
❑ Printed information (please specify) ___________________________
❑ Work modification
❑ Other 1 (please specify) ___________________________
❑ Other 2 (please specify) ___________________________
❑ Other 3 (please specify) ___________________________
❑ Referral to another health care provider (e.g. another chiropractor or physiotherapist, general practitioner, pain clinic, specialist).
If yes, please specify ___________________________

If you wish to comment on your management decisions, please do so here:

____

____

____

____

Scenario 3

A 36 year old real estate agent consults you for his low-back pain. He comes in on a very busy day at the practice and there are many patients already in the waiting room wanting to see you. The pain has been present for six weeks, starting two days after moving heavy furniture at home. The pain is described as an ache (4 to 5 out of 10). There is no radiation. He has had previous, similar episodes of low-back pain that have lasted one to two weeks. The patient has no other health concerns. The patient has seen you weekly over the last four weeks for his low-back pain and complains that it is not improving. He is frustrated with his lack of improvement and thinks something different needs to be done. He is dissatisfied that he has not already been referred for further investigation, and insists that you refer him now.

1. Which investigations would you order, undertake or recommend for this patient at this visit? *Please tick all that apply:*

☐ Lumbosacral plain x-ray
☐ Lumbar CT scan
☐ Lumbar MRI
☐ Bone scan
☐ Full spine plain x-ray
☐ Other 1 (*please specify*) ____________________________
☐ Other 2 (*please specify*) ____________________________
☐ Other 3 (*please specify*) ____________________________
☐ None

2. Which interventions would you recommend or undertake for this patient at this visit? *Please tick all that apply:*

❑ Spinal manipulation
❑ Spinal adjustment (please specify) ____________________________
❑ Mobilisation
❑ Other techniques (please circle or specify): Activator / SOT blocks / Flexion distraction / Other (specify) __________________________
❑ Massage
❑ Acupuncture / dry needling
❑ Electrotherapy (e.g. TENS, interferential, ultrasound)
❑ Thermal modalities (e.g. heat, ice)
❑ Spinal traction
❑ Bed rest for ____ days (please specify number of days)
❑ Advice to stay active
❑ Advice regarding alternate ways of moving or performing activities
❑ Advice to avoid pain provoking movements
❑ Paracetamol
❑ Non-steroidal anti-inflammatory drugs (NSAIDs)
❑ Back exercises
❑ General exercise (e.g. walking)
❑ Lumbar supports
❑ Printed information (please specify) ____________________________
❑ Work modification
❑ Other 1 (please specify) ____________________________
❑ Other 2 (please specify) ____________________________
❑ Other 3 (please specify) ____________________________
❑ Referral to another health care provider (e.g. another chiropractor or physiotherapist, general practitioner, pain clinic, specialist).
If yes, please specify ____________________________

If you wish to comment on your management decisions, please do so here:

____

____

____

____

Scenario 4

A 28 year old woman has suffered from low-back pain for a week. She has been unable to do her job managing a hospital cafeteria for this time. She walks slowly into your consultation room, holding her back and grimacing. You notice her abnormal posture. She sits with a loud groan. She says she has severe low-back pain, describing it as 9 out of 10. While anxious to return to work, she feels immobilised by the pain. There is no history of trauma. The pain is in the low-back area, without radiation. On physical examination there is marked limitation of anterior flexion and tenderness in the left paraspinal region. The neurological examination is normal with straight leg raising to 90 degrees. She has had numerous episodes of back pain in the past but thinks this is the worst episode she has ever had and is very worried that whatever is causing her problem is getting worse.

1. Which investigations would you order, undertake or recommend for this patient at this visit? *Please tick all that apply:*

- ☐ Lumbosacral plain x-ray
- ☐ Lumbar CT scan
- ☐ Lumbar MRI
- ☐ Bone scan
- ☐ Full spine plain x-ray
- ☐ Other 1 (*please specify*) ___________________________
- ☐ Other 2 (*please specify*) ___________________________
- ☐ Other 3 (*please specify*) ___________________________
- ☐ None

2. Which interventions would you recommend or undertake for this patient at this visit? *Please tick all that apply:*

- ❑ Spinal manipulation
- ❑ Spinal adjustment (please specify) ___________________________
- ❑ Mobilisation
- ❑ Other techniques (please circle or specify): Activator / SOT blocks / Flexion distraction / Other (specify) __________________________
- ❑ Massage
- ❑ Acupuncture / dry needling
- ❑ Electrotherapy (e.g. TENS, interferential, ultrasound)
- ❑ Thermal modalities (e.g. heat, ice)
- ❑ Spinal traction
- ❑ Bed rest for ____ days (please specify number of days)
- ❑ Advice to stay active
- ❑ Advice regarding alternate ways of moving or performing activities
- ❑ Advice to avoid pain provoking movements
- ❑ Paracetamol
- ❑ Non-steroidal anti-inflammatory drugs (NSAIDs)
- ❑ Back exercises
- ❑ General exercise (e.g. walking)
- ❑ Lumbar supports
- ❑ Printed information (please specify) ___________________________
- ❑ Work modification
- ❑ Other 1 (please specify) ___________________________
- ❑ Other 2 (please specify) ___________________________
- ❑ Other 3 (please specify) ___________________________
- ❑ Referral to another health care provider (e.g. another chiropractor or physiotherapist, general practitioner, pain clinic, specialist).
  If yes, please specify ___________________________

If you wish to comment on your management decisions, please do so here:

____

____

____

____

Details of the outcome measurement for the behavioural constructs

Domains	Details of outcome measurement	
	Managing patients without referral for plain x-ray	Advising patients to stay active
**Behavioural intention**		
Intention		
Generalised	3 items (e.g. "I expect to manage these patients without referring for, or taking, a plain x-ray"), each measured on a 7 point Likert scale from strongly disagree to strongly agree (1 – 7). Scores are then averaged to create a generalised behavioural intention score. Higher scores reflect greater generalised intention to manage patients without x-ray.	3 items (e.g. "I intend to advise these patients to stay active"), each measured on a 7 point Likert scale from strongly disagree to strongly agree (1 – 7). Scores are then averaged to create a generalised behavioural intention score. Higher scores reflect greater generalised intention to advise patients to stay active.
Performance	1 item ("Thinking about your next 10 patients presenting with acute uncomplicated low-back pain, how many of them will you refer for, or take, a plain x-ray?"), measured on 11 point scale (0 - 10). Higher scores reflect greater intention to x-ray.	1 item, ("Thinking about your next 10 patients presenting with acute uncomplicated low-back pain, how many of them will you advise to stay active?"), measured on 11 point scale (0 - 10). Higher scores reflect greater intention to advise patients to stay active.
**Other behavioural domains**		
Beliefs about capabilities		
	4 items (e.g. "I am confident that I can manage these patients without referring for plain x-ray"), each measured on a 7 point Likert scale from strongly disagree to strongly agree (1 – 7). Scores are then averaged to create a beliefs about capabilities score. Higher scores reflect more positive beliefs about capabilities to manage patients without x-ray.	4 items (e.g. "For me, advising these patients to stay active is easy"), each measured on a 7 point Likert scale from strongly disagree to strongly agree (1 – 7). Scores are then averaged to create a beliefs about capabilities score. Higher scores reflect more positive beliefs about capabilities.
Beliefs about consequences		
Direct attitude	5 items (e.g. "For me, managing these patients without referring for plain x-ray is..: bad practice/good practice"), measuring direct attitude using bipolar adjectives on 7 point Likert scales (1 – 7). Scores are then averaged to create a direct attitude score. Higher scores reflect more positive attitudes towards managing patients without x-ray.	5 items (e.g. "For me, advising these patients to stay active is..: the wrong thing to do/the right thing to do"), measuring direct attitude using bipolar adjectives on seven point Likert scales (1 – 7). Scores are then averaged to create a direct attitude score. Higher scores reflect more positive attitudes towards advising patients to stay active.
Behavioural beliefs	7 items (e.g. "If I manage these patients without referring for plain x-ray I feel that I am protecting them from unnecessary radiation"), each measured on 7 point Likert scales (strongly disagree to strongly agree, unlikely to likely). Scores are then averaged to create a behavioural beliefs score. Higher scores reflect more positive behavioural beliefs.	7 items (e.g. "If I advise these patients to stay active, they will improve at a faster rate"), each measured on 7 point Likert scales (strongly disagree to strongly agree, unlikely to likely). Scores are then averaged to create a behavioural beliefs score. Higher scores reflect more positive behavioural beliefs.<br><br>Practitioners' fear-avoidance beliefs about physical activity and pain measured using a modified version of the Fear-Avoidance Beliefs-Questionnaire physical activity subscale (Waddell et al: A Fear-Avoidance Beliefs Questionnaire (FABQ) and the role of fear-avoidance beliefs in chronic low back pain and disability. Pain 1993, 52:157-168); modifications available in the section "Fear avoidance beliefs questionnaire (FAB-Q) modifications". 4 items (e.g. "Physical activity makes acute uncomplicated low-back pain worse"), each measured on 7 point Likert scales (1 – 7). Scores rescaled to range between 0 – 6, then summed to create a fear-avoidance score ranging between 0 and 24. Higher scores reflect greater fear-avoidance.

Domains	Details of outcome measurement	
	Managing patients without referral for plain x-ray	Advising patients to stay active
Knowledge		
Awareness of guideline	1 item ("I am familiar with the NHMRC-endorsed evidence-based guideline for managing patients with acute uncomplicated low back pain"), measured on a 7 point Likert scale (1 – 7) from strongly disagree to strongly agree. Higher scores reflect greater awareness of guideline.	1 item ("I am familiar with the NHMRC-endorsed evidence-based guideline for managing patients with acute uncomplicated low back pain"), measured on a 7 point Likert scale (1 – 7) from strongly disagree to strongly agree. Higher scores reflect greater awareness of guideline.
Awareness of recommendation	1 item ("I am aware of the recommendation regarding use of plain x-ray from the evidence-based guideline for acute uncomplicated low back pain"), measured on a 7 point Likert scale (1 – 7) from strongly disagree to strongly agree. Higher scores reflect greater awareness of recommendation regarding x-ray.	1 item ("I am aware of the recommendation regarding advice to stay active (activation) from the evidence–based guideline for acute uncomplicated low-back pain"), measured on a 7 point Likert scale (1 – 7) from strongly disagree to strongly agree. Higher scores reflect greater awareness of recommendation about advising patients to stay active.
Understanding of recommendation	1 item ("Practising in a manner consistent with the evidence-based guideline for acute uncomplicated low-back pain means…"), asks participants to select one response from a list of 5 options. Dichotomous outcome representing understanding of recommendation created from selected response. Higher scores reflect greater understanding of recommendation regarding x-ray.	1 item ("For me, advising these patients to stay active means…"), asks participants to select one response from a list of 6 options. Dichotomous outcome representing understanding of recommendation created from selected response. Higher scores reflect greater understanding of recommendation about advising patients to stay active.
Professional role and identity		
	5 items (e.g. "Managing these patients without referring for plain x-ray is an appropriate part of my work as a physiotherapist"), each measured on a 7 point Likert scale from strongly disagree to strongly agree. Scores are then averaged to create a professional role and identity score. Higher scores reflect greater perceived professional role to manage patients without x-ray.	3 items (e.g. "It is my professional responsibility as a physiotherapist to advise these patients to stay active"), each measured on a 7 point Likert scale from strongly disagree to strongly agree. Scores are then averaged to create a professional role and identity score. Higher scores reflect greater perceived professional role to advise patients to stay active.
Social influences		
Direct (beliefs of general referents)	3 items (e.g. "People who are important to me professionally think that I should manage these patients without referring for plain x-ray"), each measured on a 7 point Likert scale from strongly disagree to strongly agree. Scores are then averaged to create a direct social influences score. Higher scores reflect greater social pressure to manage patients without x-ray.	3 items (e.g. "People whose opinions I value would approve of me advising these patients to stay active"), each measured on a 7 point Likert scale from strongly disagree to strongly agree. Scores are then averaged to create a direct social influences score. Higher scores reflect greater social pressure to advise patients to stay active.
Indirect (beliefs of specific referents)	4 items (e.g. "Other health professionals (e.g. GPs) would approve of me managing these patients without referring for plain x-ray"), each measured on a 7 point Likert scale from strongly disagree to strongly agree. Scores are then averaged to create an indirect social influences score. Higher scores reflect greater social pressure to manage patients without x-ray.	3 items (e.g. "Other physiotherapists would approve of my giving advice to stay active to these patients"), each measured on a 7 point Likert scale from strongly disagree to strongly agree. Scores are then averaged to create an indirect social influences score. Higher scores reflect greater social pressure to advise patients to stay active.
Environmental context and resources		
	1 item ("It is easy for me to refer these patients for plain x-ray because the cost is covered by Medicare"), measured on a 7 point Likert scale (1 – 7) from strongly disagree to strongly agree. Higher scores reflect greater pressure from environmental context to x-ray.	1 item ("It's difficult to find time in the consultation to advise these patients to stay active"), measured on a 7 point Likert scale (1 – 7) from strongly disagree to strongly agree. Score reversed to create environmental context score. Higher scores reflect more supportive environment in favour of advising patients to stay active.

Domains	Details of outcome measurement	
	Managing patients without referral for plain x-ray	Advising patients to stay active
Memory		
		1 item ("Sometimes I forget to advise these patients to stay active"), measured on a 7 point Likert scale from strongly disagree to strongly agree. Score reversed to create memory score. Higher scores reflect memory more supportive of advising patients to stay active.

Practice ID: Practitioner ID: Patient ID: Encounter #: **1**

Patient Encounter Form (to be completed by Physiotherapist)

**Date of encounter:**	**Start time:**	**Verbal consent obtained?:**	**Patient Date of Birth:**
______ / ______ / ______	|____:____| AM/PM	Yes ☐ No ☐ *(if No STOP collecting data here)*	___ / ___ / __ __ __ __

**Patient Sex:**	**Patient Postcode:**	**Is this the first time you have seen this patient during the data collection period?**	**Patient has LBP?**
M ☐ F ☐	|__________|	Yes ☐ No ☐	Yes ☐ No ☐ *(If Yes, check eligibility overleaf to continue below; if No, STOP here)*

**Patient meets eligibility criteria?** (see overleaf)**:**	**How long has this patient been experiencing <u>this episode</u> of LBP?:**	**Is this episode of LBP covered by Work Cover or the TAC?:**
Yes ☐ No ☐ *(If Yes, give patient an encounter form (patient-completed version); if No, STOP here)*	________ days	Yes ☐ No ☐

Which investigations have you ordered, undertaken or recommended for this patient at this visit? (Please tick all that apply)**:**

- ☐ Lumbosacral plain x-ray
- ☐ Lumbar CT scan
- ☐ Lumbar MRI
- ☐ Bone scan
- ☐ Full spine plain x-ray
- ☐ Other 1 (please specify): ______________________
- ☐ Other 2 (please specify): ______________________
- ☐ Other 3 (please specify): ______________________
- ☐ None

Which interventions did you undertake or recommend for this patient at this visit?
(The list below contains techniques that are commonly used by physiotherapists and/or chiropractors; please tick all that apply, or add to the list, as relevant to you. Note: this list is not intended to be comprehensive or guide treatment, it is simply a way of documenting techniques employed):

- ☐ Spinal manipulation
- ☐ Spinal adjustment *(please specify):* ______________________
- ☐ Mobilisation
- ☐ Other techniques *(please circle or specify)*:
  - Activator / SOT blocks / Flexion distraction
  - Other *(specify):* ______________________
- ☐ Massage
- ☐ Acupuncture / dry needling
- ☐ Electrotherapy (e.g. TENS, interferential, ultrasound)
- ☐ Thermal modalities (e.g. heat, ice)
- ☐ Spinal traction
- ☐ Bed rest for ______ days *(please specify number of days)*
- ☐ Advice to stay active
- ☐ Advice regarding alternate ways of moving or performing activities
- ☐ Advice to avoid pain provoking movements
- ☐ Paracetamol
- ☐ Non-steroidal anti-inflammatory drugs (NSAIDs)
- ☐ Back exercises
- ☐ General exercise (e.g. walking)
- ☐ Lumbar supports
- ☐ Printed information *(please specify):* ______________________
- ☐ Work modification
- ☐ Other 1 *(please specify)*: ______________________
- ☐ Other 2 *(please specify)*: ______________________
- ☐ Other 3 *(please specify)*: ______________________
- ☐ Referral to another health care provider *(e.g. another physiotherapist, chiropractor, general practitioner, pain clinic or specialist).*
  If yes, please specify: ______________________

How would you rate this patient's presenting **low back pain** today? (Please circle a number from 0 to 10)**:**

No pain 0 1 2 3 4 5 6 7 8 9 10 *Severe pain*

How would you rate this patient's general health? (please circle a number from 0 to 10):

Poor health 0 1 2 3 4 5 6 7 8 9 10 *Excellent health*

Eligibility criteria

The patient must meet all of the following inclusion criteria (i.e. a 'YES' must be achieved for all):

- the **current** episode of LBP has been present for **less than 3 months** *(this includes recurrent episodes of LBP that are less than 3 months duration; also known as 'serial acute LBP', 'acute flare-ups of LBP' or 'repeated but discrete episodes of acute LBP')*
- they understand and read English
- they are 18 years of age or older
- they haven't undergone previous spinal surgery
- they don't have radicular leg pain present (defined as leg pain that is described as shooting, lancinating, or electric in quality, extends below the knee, has a dermatomal distribution and may be associated with paraesthesia)
- they don't have fracture of the spine, cancer or high fever
- (females only) they are not pregnant

Patient Encounter Form (to be completed by patient)

There are a range of tests and treatments that physiotherapists may use when treating people with back pain. The purpose of this brief checklist is to ask you about the tests and treatments that your physiotherapist has given to you today. It is not necessary for your physiotherapist to have delivered all these procedures. It also asks you some basic information about you (e.g. your gender etc).

**Today's date:**	**Are you male or female?:**	**What is your date of birth?:**	**What is your postcode:**
______ / ______ / ______	M ☐ F ☐	___ / ___ / __ __ __ __	|__________|

**How long have you had your current episode/flare-up of back pain?:**	**Have you had previous episodes/flare-ups of back pain?:**	**Is your current episode/ flare-up of back pain covered by Work Cover or the TAC?:**
__________ days<br>or<br>__________ weeks	Yes ☐ No ☐<br>*If YES, how many episodes of back pain have you had in the last 12 months?*<br>__________ **(number of episodes/flare-ups)**	Yes ☐ No ☐

What tests, *if any*, **were ordered or undertaken by your physiotherapist at this visit for your current episode/flare-up of back pain?** *[The list below contains tests that may or may not be relevant to you; please tick all that apply or use the 'other' category to describe anything not covered in the list provided]*

- ☐ None
- ☐ X-ray
- ☐ Physical examination
- ☐ Other radiological tests (e.g. CT scan, MRI scan, bone scan, ultrasound)
- ☐ Other *(please specify)*: ______________________________

What treatment(s) and advice did your physiotherapist recommend or give you at this visit for your current episode/ flare-up of back pain? *[The list below contains treatments that are sometimes used although they may not be relevant for or needed by you; please tick all that apply or use 'other' categories to describe anything not covered by the list provided]*

- ☐ Bed rest for __________ days *(please specify number of days)*
- ☐ Medication
- ☐ Manual therapy *(such as massage, movement of my back by my physiotherapist etc)*
- ☐ Exercises for my back
- ☐ Advice to stay active
- ☐ Other advice *(please specify what about)* ______________________________
  ____
- ☐ Work modification
- ☐ Printed handout
- ☐ Other *(please specify)* ______________________________
  ____
- ☐ Referral to another health care provider (e.g. another physiotherapist, chiropractor, general practitioner, pain clinic, specialist). *If yes, please specify to who* ______________________________

**Have you previously received an x-ray for this episode/ flare-up of your back pain?:**	**Have you previously received any other radiological tests (e.g. CT scan, MRI, bone scan) for this episode/flare-up of your back pain?:**
Yes ☐ No ☐	Yes ☐ No ☐
If YES, who ordered this x-ray?<br>*Physiotherapist* ☐ *Other* ☐ *(please specify)*:______________	*If YES, who ordered this x-ray?*<br>*Physiotherapist* ☐ *Other* ☐ *(please specify)*:______________

How would you rate your presenting **low back pain** today? (Please circle a number from 0 to 10)**:**

No pain 0 1 2 3 4 5 6 7 8 9 10 *Severe pain*

We would also like to invite you to participate in two follow-up activities (completion of one survey mailed to you at 3 months following the onset of your episode/flare-up of low-back pain; and permission for a member of the research team to access your clinic file, located at your practitioner's office, to review the management provided by your practitioner for your back pain). Participation in these additional activities will take approximately 15 minutes of your time. If you would like to participate in these please complete the details below. Thank you for your participation in this study; it is highly appreciated.

CONSENT TO ADDITIONAL ACTIVITIES

I agree to take part in the additional activities of the research project. I have had the project explained to me, and I have read the Explanatory Statement, which I keep for my records. I understand that agreeing to take part means that (please tick below):

☐ I agree to allow the research team to mail me 1 survey to complete (at 3 months) that I can complete and return using the reply-paid envelope provided

☐ I agree to allow my clinic file (at my practitioner's office) to be made available for this research

I understand that my participation is voluntary, that I can choose not to participate in part or all of the project, and that I can withdraw prior to my practitioner entering my information on the encounter form during my visit. I understand that any data that the researcher collects from me or my clinic file for use in reports or published findings will not, under any circumstances, contain names or identifying characteristics. I understand that any information I provide is confidential, and that no information that could lead to the identification of any individual will be disclosed in any reports on the project, or to any other party. I understand that data from my clinic file will be kept in secure storage and accessible only to the research team. I also understand that the data will be destroyed after a 15 year period unless I consent to it being used in future research. This consent will remain with the Monash University researcher for their records.

Please also tick one box below:

☐ The information I provide can be used in further research projects which have ethics approval as long as my name and contact information is removed before it is given to them

☐ The information I provide cannot be used by other researchers without asking me first

☐ The information I provide cannot be used except for this project

PLEASE COMPLETE THE INFORMATION BELOW

Name *(first name, surname)**: ______________________________

Mailing address *(number and street, suburb, postcode)** ______________________________

____

Signature*: ______________________________

Date*: ______________________________

Contact phone number*(s)**: ______________________________

** Fields indicated with asterisks are compulsory*

Please return this form in the envelope provided to the locked box at reception. Please ask the receptionist if you cannot locate it and she/he will assist you. If you would prefer to send the form directly to the research team, please ask reception for a reply-paid (postage-paid) envelope and you can mail this form directly to us.

QUESTIONNAIRES FOR PATIENTS

Office use only : | PERSON ID : | | CLINIC ID: | |

Management of people with acute low-back pain

Thank you for agreeing to complete this survey.

The aim of the survey is to provide information about the nature of your back pain and the impact it is having (or has had in the past) on your life.

This survey contains five sections and will take approximately 15 minutes to complete.

Please read each question carefully. We are interested in the progress of your low-back pain and general health, but there are no correct or incorrect responses. Please answer all of the questions as best you can.

All information that you provide will be confidential. No information that could lead to the identification of any individuals will be disclosed in any reports or to any other party. Completed surveys will be kept in a locked filing cabinet at the Monash Institute of Health Services Research for a period of five years and will then be destroyed.

Thank you very much for your participation

Professor Sally Green, Professor Jenny Keating and Dr Bruce Walker
on behalf of the ALIGN research team
Institute of Health Services Research and School of Primary Health Care, Monash University,
and School of Chiropractic and Sports Science, Murdoch University

If you have any questions or would like more information please contact:
Dr Denise O'Connor (Project Manager), Monash Institute of Health Services Research
T: 1300 798 527
F: (03) 9594-7570
E: align.mihsr@med.monash.edu.au

PART A

1 How would you rate your back pain on a 0-10 scale at the present time, that is right now, where 0 is ‘no pain’ and 10 is ‘pain as bad as it could be’? [*please circle a number from 0 to 10*]

No pain	0	1	2	3	4	5	6	7	8	9	10	*Pain as bad as it could be*

2 In the past week, how intense was your worst pain rated on a 0-10 scale where 0 is ‘no pain’ and 10 is ‘pain as bad as it could be’? [*please circle a number from 0 to 10*]

No pain	0	1	2	3	4	5	6	7	8	9	10	*Pain as bad as it could be*

3 In the past week, on the average, how intense was your pain rated on a 0-10 scale where 0 is ‘no pain’ and 10 is ‘pain as bad as it could be’? [*please circle a number from 0 to 10*]

No pain	0	1	2	3	4	5	6	7	8	9	10	*Pain as bad as it could be*

4 In the past week, how intense was your least pain rated on a 0-10 scale where 0 is ‘no pain’ and 10 is ‘pain as bad as it could be’? [*please circle a number from 0 to 10*]

No pain	0	1	2	3	4	5	6	7	8	9	10	*Pain as bad as it could be*

5 How long have you been experiencing this particular episode/flare-up of **low back pain**? [*please specify in days*]

________ Day(s)

6 Have you received an x-ray for this episode/flare-up of your back pain? [*please tick one box*]

❑ Yes ❑ No

If yes, who referred you for this x-ray?

❑ Chiropractor

❑ Physiotherapist

❑ Other ______________________________

7 Have you received a CT-scan, MRI or a bone scan for this episode/flare-up of your back pain? [*please tick one box*]

❑ Yes ❑ No

If yes, who referred you for this CT/MRI/bone scan?

❑ Chiropractor

❑ Physiotherapist

❑ Other ______________________________

PART B

Here are some of the things which other people have told us about their pain. For each statement please circle the number from 1 to 7 to say how much physical activities (such as bending, lifting, walking or driving) affect, or would affect, your back pain:

**1**	Physical activity makes my pain worse	*Completely disagree*	1	2	3	4	5	6	7	*Completely agree*
**2**	Physical activity might harm my back	*Completely disagree*	1	2	3	4	5	6	7	*Completely agree*
**3**	I should not do physical activities which (might) make my pain worse	*Completely disagree*	1	2	3	4	5	6	7	*Completely agree*
**4**	I cannot do physical activities which (might) make my pain worse	*Completely disagree*	1	2	3	4	5	6	7	*Completely agree*

PART C

When your back hurts, you may find it difficult to do some things you normally do.

This list contains some sentences that people have used to describe themselves when they have back pain. When you read them, you may find that some stand out because they describe you **today**. As you read the list, think of yourself **today**. When you read a sentence that describes you today, put a tick against it. If the sentence does not describe you, then leave the box blank and go on to the next one. Remember, only tick the sentence if you are sure that it describes you **today**.

- ❑ I stay at home most of the time because of my back.
- ❑ I change positions frequently to try and get my back comfortable.
- ❑ I walk more slowly than usual because of my back.
- ❑ Because of my back, I am not doing any of the jobs that I usually do around the house.
- ❑ Because of my back, I use a handrail to get upstairs.
- ❑ Because of my back, I lie down to rest more often.
- ❑ Because of my back, I have to hold on to something to get out of an easy chair.
- ❑ Because of my back, I try to get other people to do things for me.
- ❑ I get dressed more slowly than usual because of my back.
- ❑ I only stand up for short periods of time because of my back.
- ❑ Because of my back, I try not to bend or kneel down.
- ❑ I find it difficult to get out of a chair because of my back.
- ❑ My back is painful almost all the time.
- ❑ I find it difficult to turn over in bed because of my back.
- ❑ My appetite is not very good because of my back pain.
- ❑ I have trouble putting on my socks (or stockings) because of the pain in my back.
- ❑ I only walk short distances because of my back pain.
- ❑ I sleep less well because of my back.
- ❑ Because of my back pain, I get dressed with help from someone else.
- ❑ I sit down for most of the day because of my back.
- ❑ I avoid heavy jobs around the house because of my back.
- ❑ Because of my back pain, I am more irritable and bad tempered with people than usual.
- ❑ Because of my back, I go upstairs more slowly than usual.
- ❑ I stay in bed most of the time because of my back.

PART D

The following questions are about your health **during the last week**. Please read them carefully, and tick the box next to the answer that best describes you **during the last week**.

**1**	Concerning my use of prescribed medicines:<br><br>[*please tick one box*]	❑ I do not or rarely use any medicines at all.<br>❑ I use one or two medicinal drugs regularly.<br>❑ I need to use three or four medicinal drugs regularly.<br>❑ I use five or more medicinal drugs regularly.
**2**	To what extent do I rely on medicines or a medical aid (NOT glasses or a hearing aid)?<br>(*for example: walking frame, wheelchair, prosthesis etc.)*<br><br>[*please tick one box*]	❑ I do not use any medicines and/or medical aids.<br>❑ I occasionally use medicines and/or medical aids.<br>❑ I regularly use medicines and/or medical aids.<br>❑ I have to constantly take medicines or use a medical aid.
**3**	Do I need regular medical treatment from a doctor or other health professional?<br><br>[*please tick one box*]	❑ I do not need regular medical treatment.<br>❑ Although I have some regular medical treatment, I am not dependent on this.<br>❑ I am dependent on having regular medical treatment.<br>❑ My life is dependent upon regular medical treatment.
**4**	Do I need any help looking after myself?<br><br>[*please tick one box*]	❑ I need no help at all.<br>❑ Occasionally I need some help with personal care tasks.<br>❑ I need help with the more difficult personal care tasks.<br>❑ I need daily help with most or all personal care tasks.
**5**	When doing household tasks:<br><br>(*for example, preparing food, gardening, using the video recorder, radio, telephone or washing the car*)<br><br>[*please tick one box*]	❑ I need no help at all.<br>❑ Occasionally I need some help with household tasks.<br>❑ I need help with the more difficult household tasks.<br>❑ I need daily help with most or all household tasks.
**6**	Thinking about how easily I can get around my home and community:<br><br>[*please tick one box*]	❑ I get around my home and community by myself without any difficulty.<br>❑ I find it difficult to get around my home and community by myself.<br>❑ I cannot get around the community by myself, but I can get around my home with some difficulty.<br>❑ I cannot get around either the community or my home by myself.
**7**	Because of my health, my relationships (*for example: with my friends, partner or parents*) generally:<br><br>[*please tick one box*]	❑ Are very close and warm.<br>❑ Are sometimes close and warm.<br>❑ Are seldom close and warm.<br>❑ I have no close and warm relationships.

**8**	Thinking about my relationships with other people:<br><br>[*please tick one box*]	❑ I have plenty of friends, and am never lonely.<br>❑ Although I have friends, I am occasionally lonely.<br>❑ I have some friends, but am often lonely for company.<br>❑ I am socially isolated and feel lonely.
**9**	Thinking about my health and my relationship with my family:<br><br>[*please tick one box*]	❑ My role in the family is unaffected by my health.<br>❑ There are some parts of my family role I cannot carry out.<br>❑ There are many parts of my family role I cannot carry out.<br>❑ I cannot carry out any part of my family role.
**10**	Thinking about my vision, including when using my glasses or contact lenses if needed:<br><br>[*please tick one box*]	❑ I see normally.<br>❑ I have some difficulty focusing on things, or I do not see them sharply (*for example: small print, a newspaper, or seeing objects in the distance*).<br>❑ I have a lot of difficulty seeing things. My vision is blurred (*for example: I can see just enough to get by with*).<br>❑ I only see general shapes, or am blind (*for example: I need a guide to move around*).
**11**	Thinking about my hearing, including using my hearing aid if needed:<br><br>[*please tick one box*]	❑ I hear normally.<br>❑ I have some difficulty hearing, or I do not hear clearly (*for example: I ask people to speak up, or turn up the TV or radio volume*).<br>❑ I have difficulty hearing things clearly (*for example: often I do not understand what is said. I usually do not take part in conversations because I cannot hear what is said*).<br>❑ I hear very little indeed (*for example: I cannot fully understand loud voices speaking directly to me*).
**12**	When I communicate with others:<br><br>(*for example: by talking, listening, writing or signing*)<br><br>[*please tick one box*]	❑ I have no trouble speaking to them or understanding what they are saying.<br>❑ I have some difficulty being understood by people who do not know me. I have no trouble understanding what others are saying to me.<br>❑ I am only understood by people who know me well. I have great trouble understanding what others are saying to me.<br>❑ I cannot adequately communicate with others.
**13**	If I think about how I sleep:<br><br>[*please tick one box*]	❑ I am able to sleep without difficulty most of the time.<br>❑ My sleep is interrupted some of the time, but I am usually able to go back to sleep without difficulty.<br>❑ My sleep is interrupted most nights, but I am usually able to go back to sleep without difficulty.<br>❑ I sleep in short bursts only. I am awake most of the night.<br>.
**14**	Thinking about how I generally feel:<br><br>[*please tick one box*]	❑ I do not feel anxious, worried or depressed.<br>❑ I am slightly anxious, worried or depressed.<br>❑ I feel moderately anxious, worried or depressed.<br>❑ I am extremely anxious, worried or depressed.

**15**	How much pain or discomfort do I experience?<br><br>[*please tick one box*]	❑ None at all.<br>❑ I have moderate pain.<br>❑ I suffer from severe pain.<br>❑ I suffer unbearable pain.

PART E

Please respond to the following items, thinking about your use of health services **over the last 3 months**.

**1**	Have you received an x-ray in the last 3 months?<br>[*please tick one box*]	❑ Yes ❑ No<br><br>If yes, how many times have you attended radiology for an x-ray in the last 3 months?___________
**2**	Have you been hospitalised in the last 3 months?<br>[*please tick one box*]	❑ Yes ❑ No<br><br>If yes, how many times have you been hospitalised in the last 3 months?___________
**3**	Have you visited casualty/emergency in the last 3 months?<br>[*please tick one box*]	❑ Yes ❑ No<br><br>If yes, how many times have you visited casualty/emergency in the last 3 months?___________
**4**	Have you visited an outpatient or day clinic in the last 3 months?<br>[*please tick one box*]	❑ Yes ❑ No<br><br>If yes, how many times have you visited an outpatient or day clinic in the last 3 months?_________
**5**	Have you visited any GP in the last 3 months?<br>[*please tick one box*]	❑ Yes ❑ No<br><br>If yes, how many times have you visited a GP in the last 3 months?_________
**6**	Have you visited a medical/surgical specialist in the last 3 months?<br>[*please tick one box*]	❑ Yes ❑ No<br><br>If yes, how many times have you visited a specialist in the last 3 months?________
**7**	Have you visited any physiotherapist in the last 3 months?<br>[*please tick one box*]	❑ Yes ❑ No<br><br>If yes, how many times have you consulted a physiotherapist in the last 3 months?_________
**8**	Have you visited any chiropractor in the last 3 months?<br>[*please tick one box*]	❑ Yes ❑ No<br><br>If yes, how many times have you visited a chiropractor in the last 3 months?_________
**9**	Have you visited any osteopath in the last 3 months?<br>[*please tick one box*]	❑ Yes ❑ No<br><br>If yes, how many times have you visited an osteopath in the last 3 months?_________

**10**	Have you visited any other health provider (other than a physiotherapist, chiropractor, osteopath) in the last 3 months?<br>[*please tick one box*]	❑ Yes ❑ No<br><br>If yes, how many times have you visited this type of other health provider in the last 3 months?__________
**11**	Have you visited any other health provider (other than the 'other health provider' above) in the last 3 months?<br>[*please tick one box*]	❑ Yes ❑ No<br><br>If yes, how many times have you visited this type of other health provider in the last 3 months?__________
**12**	Have you used any prescription or over-the-counter medications in the last 3 months?<br>[*please tick one box*]	❑ Yes ❑ No<br><br>If yes, how many different medications have you used in the last 3 months?__________
**13**	Have you used any prescription or over-the-counter pain relievers in the last 3 months?<br>[*please tick one box*]	❑ Yes ❑ No<br><br>If yes, on how many days have you taken pain relievers in the last 3 months?__________
**14**	Have you used any prescription or over-the-counter sleeping medications in the last 3 months?<br>[*please tick one box*]	❑ Yes ❑ No<br><br>If yes, on how many days have you taken sleeping medications in the last 3 months?__________
**15**	What is your usual main activity?<br>[*please tick one box*]	❑ Full-time student<br>❑ Part-time student<br>❑ Employed<br>❑ Unemployed<br>❑ Retired
**16**	How many hours would you usually spend on your main activity in a week?<br>[*please tick one box*]	❑ 0 hours<br>❑ 1-15 hours<br>❑ 16-24 hours<br>❑ 25-34 hours<br>❑ 35-40 hours<br>❑ 41-48 hours<br>❑ 49 hours or more
**17**	Have you spent time away from your usual main activity due to illness or to attend treatment in the last 3 months<br>[*please tick one box*]	❑ Yes ❑ No<br><br>If yes, how many full days away from usual main activity due to illness in the last 3 months?__________<br><br>And how many hours away from usual main activity to attend treatment in the last 3 months?__________

Thank you very much for completing this survey

FEAR AVOIDANCE BELIEFS QUESTIONNAIRE (FAB-Q) MODIFICATIONS

At the practitioner level, the original FAB-Q has been modified so that it can apply to practitioners. The following changes have been made to the instructions and to the four physical activity related items from the original questionnaire:

Original	Modifications for practitioners FAB-Q in the ALIGN study
Instructions: Here are some of the things which other patients have told us about their pain. For each statement please circle any number from 0 to 6 to say how much physical activities (such as bending, lifting, walking or driving) affect or would affect back pain	Modified instructions: Here are some of the things which people have told us about their acute low-back pain. For each statement please circle any number from 1 to 7 to say how much physical activities (such as bending, lifting, walking or driving) affect or would affect back pain.
Item 1: Physical activity makes my pain worse	Physical activity makes acute uncomplicated low-back pain worse
Item 2: Physical activity might harm my back	Physical activity might harm people with acute uncomplicated low-back pain
Item 3: I should not do physical activities which (might) make my pain worse	People with acute uncomplicated low-back pain should not do physical activities which (might) make their pain worse
Item 4: I cannot do physical activities which (might) make my pain worse	People with acute uncomplicated low-back pain cannot do physical activities which (might) make their pain worse

CONTENT FOR COLLECTION IN FILE AUDIT

Some interventions, particularly the guideline-recommended intervention of providing 'advice to stay active', are not routinely recorded in the patient's file. Therefore, we have adopted the approach of using both sources (i.e. from encounter forms and file audit) to document the investigations and interventions provided by participating practitioners. Where there is a discrepancy between encounter forms and file audit, we will accept evidence of the investigation/intervention occurring from either source but note the frequency and nature of the discrepancies in our final report. The variables for documentation in the file audit are listed below.

1. Is there evidence in the file that the patient was referred for plain x-ray at any time during their care for this episode of LBP? (Y/N)
2. If yes, what was the date of the referral for x-ray or date of x-ray undertaken? (date)
3. Is there a report for a plain x-ray? (Y/N)
4. If yes, what is the date of the report? (date)
5. Is there evidence in the file that the patient was referred for other types of imaging, e.g. CT scan, MRI, bone scan? (Y/N)
6. If yes, what was the date of the referral for other types of imaging, e.g. CT scan, MRI, bone scan or date of other types of imaging, e.g. CT scan, MRI, bone scan undertaken? (date)
7. Is there a report for other types of imaging, e.g. CT scan, MRI, bone scan? (Y/N)
8. If yes, what is the date of the report? (date)
9. Extra notes relevant to investigations and/or interventions undertaken for this episode of LBP.
