# Supplementary material for: Improving the care for people with acute low-back pain by allied health professionals (the ALIGN trial): A cluster randomised trial protocol
Source: Implement Sci. 2010 Nov 10;5:86. doi: 10.1186/1748-5908-5-86 (PMC2994785; doi:10.1186/1748-5908-5-86)
Supplement: Additional file 4 — ALIGN sample size calculations. This file includes details of the assumptions and calculations in estimating the likely width of the 95% CI for the risk difference and the log odds ratio, for the primary practitioner outcome, x-ray referral. [file 1748-5908-5-86-S4.PDF]

Sample size calculations for the ALIGN cluster randomised trial

Justification for the original sample size calculation is made in the trial protocol. In this additional file, we provide details of the assumptions and calculations in estimating the likely width of the 95% confidence interval (CI) for the estimators, the risk difference and the log odds ratio, for the primary practitioner outcome, x-ray referral. Table 1 contains the likely widths of the 95% CIs for these estimators assuming a range of values of the sample size parameters (cluster (practice) size, coefficient of variation (CV), design effect, and control and intervention group x-ray referral rates). Justification for the selected values of the parameters follows.

Number of practices: At baseline we randomised 210 practices (133 physiotherapy and 77 chiropractic practices); assuming 20% attrition, we estimate that 168 practices will complete the data collection (completion of patient encounter forms over a two week period).

Cluster size: From an analysis of the baseline data collected in the practitioner questionnaire, on average, 41 acute low-back pain (LBP) patients (standard deviation = 40.2) are treated in a two week period. Sample size calculations in Table 1 are therefore based around this average, ranging from 15 to 40 patient participants per cluster.

Coefficient of variation: A CV, defined as the ratio of the standard deviation of cluster sizes to the mean cluster size, was calculated from the baseline data as 0.97. We have also investigated the affect of two other CVs (0.6, 0.7) on the width of the CIs. These CVs have been observed in general practice in the United Kingdom (UK) [1].

Design effect: When all clusters are of equal size, the design effect is a function of cluster size and the intra-cluster correlation coefficient (ICC). When the cluster sizes vary, the design effect becomes a function of the average cluster size, the ICC, and variation in cluster size. We have based our sample size calculations on the latter design effect, using equation (2) in Eldridge et al [1], which allows for expected variation in cluster size using a CV. For comparison, we also present the design effect assuming all clusters are of equal size (column 3, Table 1). Sample size calculations in Table 1 assume an ICC of 0.10. Empirical research has suggested ICCs of this magnitude for process variables, such as x-ray referral, in primary care [2].

Control group x-ray referral rates: We have assumed two control group x-ray referral rates. The first control group x-ray referral rate has been calculated from the assumptions we made in our original sample size calculation (see the trial protocol for details). In brief, we assumed an x-ray referral rate of 28% and 67% for physiotherapists and chiropractors respectively. The weighted average of these rates (assuming 106 physiotherapy and 62 chiropractic practices at follow-up) is 35%. Assuming there is an 8% improvement in the control group [3], at follow-up we predict a control group x-ray referral rate of 27%.

The second control group x-ray referral rate has been estimated from a survey of Australian primary care physicians (undertaken in 2000), investigating non-specific LBP assessment methods [4]. Practitioners were asked to select how frequently they would use imaging, including x-ray. From the presented data (Table 4 [4]), we have

estimated x-ray usage rates for physiotherapists and chiropractors to be 43% and 57%, respectively. The weighted average of these rates is 48%. Assuming that x-ray usage rates reflect x-ray referral rates, and that there will be an 8% improvement in the control group [3], at follow-up we predict a control group x-ray referral rate of 40%.

Intervention group x-ray referral rates: We have calculated the widths of the 95% CIs assuming a difference of 10% and 15% between the intervention and control groups at follow-up. However, as can be seen from the widths of the CIs in Table 1, the standard errors of the estimates are not affected to any important degree by the intervention group rates.

Summary

If the assumptions underlying the sample size calculations are reasonable, the width of the 95% CI for the observed difference in x-ray referral rates between groups at follow-up is likely to be in the range of ±5% to ±7%. On the log odds scale, this is equivalent to a range of ±0.26 to ±0.41.

Table 1 – Likely widths of 95% confidence intervals for the ALIGN trial under various assumptions

Patients/ practice	Total sample size (N)[3]	$DE_{(no\ adj.)}$[4]	$DE_{(adj)}$[5]	ESS (using $DE_{(adj)}$)[6]		**Difference in rates (RD)**[7] Width of 95% confidence interval for a difference in rates				**Log odds ratio (OR)**[8] Width of 95% confidence interval for the difference in log odds			
					Int. group rate (p_{int})	17%	12%	30%	25%	17%	12%	30%	25%
					Control group rate (p_{ctrl})	27%	27%	40%	40%	27%	27%	40%	40%
					Estimate (RD or ln(OR))	-10%	-15%	-10%	-15%	-0.59	-1.00	-0.44	-0.69
					(OR)					(0.55)	(0.37)	(0.64)	(0.50)
CV = 0.97[1]													
15	2520	2.4	3.8	661		±6.3%	±5.9%	±7.2%	±7.0%	±0.38	±0.41	±0.32	±0.33
20	3360	2.9	4.8	703		±6.1%	±5.7%	±7.0%	±6.8%	±0.36	±0.40	±0.31	±0.32
25	4200	3.4	5.8	730		±6.0%	±5.6%	±6.9%	±6.7%	±0.36	±0.39	±0.31	±0.32
30	5040	3.9	6.7	750		±5.9%	±5.6%	±6.8%	±6.6%	±0.35	±0.39	±0.30	±0.31
40	6720	4.9	8.7	776		±5.8%	±5.5%	±6.7%	±6.5%	±0.35	±0.38	±0.30	±0.31
CV = 0.70[2]													
15	2520	2.4	3.1	804		±5.7%	±5.4%	±6.6%	±6.4%	±0.34	±0.37	±0.29	±0.30
20	3360	2.9	3.9	866		±5.5%	±5.2%	±6.3%	±6.2%	±0.33	±0.36	±0.28	±0.29
25	4200	3.4	4.6	908		±5.3%	±5.1%	±6.2%	±6.0%	±0.32	±0.35	±0.27	±0.28
30	5040	3.9	5.4	939		±5.3%	±5.0%	±6.1%	±5.9%	±0.32	±0.34	±0.27	±0.28
40	6720	4.9	6.9	980		±5.1%	±4.9%	±5.9%	±5.8%	±0.31	±0.34	±0.26	±0.27
CV = 0.60[2]													
15	2520	2.4	3.0	831		±5.6%	±5.3%	±6.4%	±6.3%	±0.34	±0.37	±0.29	±0.30
20	3360	2.9	3.7	897		±5.4%	±5.1%	±6.2%	±6.0%	±0.32	±0.35	±0.28	±0.29
25	4200	3.4	4.5	942		±5.3%	±5.0%	±6.1%	±5.9%	±0.31	±0.34	±0.27	±0.28
30	5040	3.9	5.2	975		±5.2%	±4.9%	±6.0%	±5.8%	±0.31	±0.34	±0.27	±0.27
40	6720	4.9	6.6	1020		±5.0%	±4.8%	±5.8%	±5.7%	±0.30	±0.33	±0.26	±0.27

CV = Coefficient of Variation; ESS = Effective Sample Size; DE = Design Effect

Widths of confidence intervals are calculated assuming an ICC of 0.10.

No adjustment has been made in the sample size calculations for stratification or confounding variables.

[1] CV calculated from self-reported number of acute LBP patients treated per two weeks (baseline practitioner questionnaire).

[2] CV from Eldridge et al [1]; observed in UK general practice.

[3] Total sample size is calculated assuming at follow-up there will be 168 practices (allows for 20% attrition in practices).

[4] Design effect calculated with no adjustment made for the variation in cluster size: $DE_{(no\ adj.)} = 1 + (m - 1)\rho$, where m is the cluster size (assuming all clusters are equal in size) and ρ is the ICC [1].

[5] Design effect calculated with adjustment for unequal cluster sizes: $DE_{(adj.)} = 1 + \{(CV^2 + 1)n - 1\}\,\rho$, where n is the average cluster size, CV is the coefficient of variation, and ρ is the ICC [1].

[6] The effective sample size is calculated as: $N/DE_{(adj)}$.

[7] The standard error of the difference in rates is calculated as: $SE(p_{int} - p_{ctrl}) = \sqrt{\frac{p_{int.}(1-p_{int.})}{ESS_{int}} + \frac{p_{ctrl}(1-p_{ctrl})}{ESS_{ctrl}}}$.

[8] The standard error of the log odds ratio is calculated as: $SE(\log OR) = \sqrt{\frac{1}{a} + \frac{1}{b} + \frac{1}{c} + \frac{1}{d}}$ where a, b, c, d, are the frequencies of effective sample size found in the cross-tabulation table of intervention group by x-ray referral [5].

References

1. Eldridge SM, Ashby D, Kerry S: **Sample size for cluster randomized trials: effect of coefficient of variation of cluster size and analysis method.** *Int J Epidemiol* 2006, **35:**1292-1300.
2. Campbell M, Grimshaw J, Steen N: **Sample size calculations for cluster randomised trials. Changing Professional Practice in Europe Group (EU BIOMED II Concerted Action).** *J Health Serv Res Policy* 2000, **5:**12-16.
3. Grimshaw J, Thomas R, MacLennan G, Fraser C, et al: **Effectiveness and efficiency of guideline dissemination and implementation strategies.** *Health Technol Assess* 2004, **8:**1–84.
4. Kent PM, Keating JL, Taylor NF: **Primary care clinicians use variable methods to assess acute nonspecific low back pain and usually focus on impairments.** *Man Ther* 2009, **14:**88-100.
5. Bland JM, Altman DG: **Statistics notes: The odds ratio.** *BMJ* 2000, **320:**1468.
